# *trans*-Translation inhibitors bind to a novel site on the ribosome and clear *Neisseria gonorrhoeae* in vivo

**DOI:** 10.1038/s41467-021-22012-7

**Published:** 2021-03-19

**Authors:** Zachary D. Aron, Atousa Mehrani, Eric D. Hoffer, Kristie L. Connolly, Pooja Srinivas, Matthew C. Torhan, John N. Alumasa, Mynthia Cabrera, Divya Hosangadi, Jay S. Barbor, Steven C. Cardinale, Steven M. Kwasny, Lucas R. Morin, Michelle M. Butler, Timothy J. Opperman, Terry L. Bowlin, Ann Jerse, Scott M. Stagg, Christine M. Dunham, Kenneth C. Keiler

**Affiliations:** 1 Microbiotix, Inc. One Innovation Dr., Worcester, MA USA; 2 Department of Chemistry and Biochemistry, Florida State University, Tallahassee, FL USA; 3 Department of Biochemistry and Emory Antibiotic Resistance Center, Emory University School of Medicine, Atlanta, GA USA; 4 Department of Microbiology and Immunology, Uniformed Services University, Bethesda, MD USA; 5 Molecular & Systems Pharmacology Graduate Program, Emory University, Atlanta, GA USA; 6 Department of Biochemistry & Molecular Biology, Penn State University, University Park, PA USA; 7 Institute of Molecular Biophysics, Florida State University, Tallahassee, FL USA

**Keywords:** Cryoelectron microscopy, Ribosome

## Abstract

Bacterial ribosome rescue pathways that remove ribosomes stalled on mRNAs during translation have been proposed as novel antibiotic targets because they are essential in bacteria and are not conserved in humans. We previously reported the discovery of a family of acylaminooxadiazoles that selectively inhibit *trans*-translation, the main ribosome rescue pathway in bacteria. Here, we report optimization of the pharmacokinetic and antibiotic properties of the acylaminooxadiazoles, producing MBX-4132, which clears multiple-drug resistant *Neisseria gonorrhoeae* infection in mice after a single oral dose. Single particle cryogenic-EM studies of non-stop ribosomes show that acylaminooxadiazoles bind to a unique site near the peptidyl-transfer center and significantly alter the conformation of ribosomal protein bL27, suggesting a novel mechanism for specific inhibition of *trans*-translation by these molecules. These results show that *trans*-translation is a viable therapeutic target and reveal a new conformation within the bacterial ribosome that may be critical for ribosome rescue pathways.

## Abstract (web summary)

Antibiotic-resistant bacterial pathogens pose a substantial threat to human health. Here, aided by structural analyses, the authors describe the molecular mechanism behind the activity of a series of compounds that inhibit trans-translation and are effective in eradicating *N. gonorrhoeae* infection in mice.

## Introduction

Antibiotic-resistant bacterial pathogens pose a substantial threat to human health and are projected to cause up to 10 million deaths per year by 2050 if new antibiotics are not developed^1^. Among the five most dangerous “Urgent Threats” identified by the CDC is drug-resistant *Neisseria gonorrhoeae*, which infects >500,000 people per year in the US^2^. New antibiotics must be developed to treat these drug-resistant infections. Bacterial ribosome rescue pathways have been proposed as potential antibiotic targets because these pathways are essential in bacterial pathogens but are highly dissimilar from cytoplasmic mechanisms to disassemble non-stop ribosomes in eukaryotes^3^.

Bacterial ribosomes frequently stall at the 3′ end of mRNAs lacking an in-frame stop codon due to physical or nucleolytic damage to the mRNA or premature termination of transcription^4^. These “non-stop” ribosomes must be rescued to maintain protein synthesis capacity and cell viability^4^. All bacterial species that have been studied use *trans*-translation as the primary ribosome rescue pathway. During *trans*-translation, transfer-messenger RNA (tmRNA) and the protein SmpB recognize non-stop ribosomes and use tRNA-like and mRNA-like properties of tmRNA to add a short sequence to the nascent polypeptide and terminate translation at a stop codon within tmRNA^4^. In some bacteria, including *N. gonorrhoeae*, tmRNA and SmpB are essential^5,6^. Other species have an alternative ribosome rescue factor (ArfA, ArfB, ArfT, or BrfA) that acts as a backup system for rescuing ribosomes when *trans*-translation activity is not sufficient^7–10^. Deletions of the alternative ribosome rescue factors and tmRNA or SmpB are synthetically lethal, indicating that at least one mechanism for ribosome rescue is required for bacterial viability^5^.

A family of acylaminooxadiazoles identified in a high-throughput screen for inhibitors of *trans*-translation in *Escherichia coli* has broad-spectrum antibiotic activity in vitro^3,11^. Experiments in *E. coli* and *Mycobacterium smegmatis* showed that KKL-2098, a cross-linkable acylaminooxadiazole derivative, bound 23S rRNA near the peptidyl-transfer center (PTC), suggesting that these molecules inhibit *trans*-translation by binding the ribosome^11^. Based on these data, we sought to optimize the acylaminooxadiazole activity against *N. gonorrhoeae* and establish the basis for selective inhibition of *trans*-translation.

## Results

### Development of a *trans*-translation inhibitor that is active in animals

**Fig. 1:**
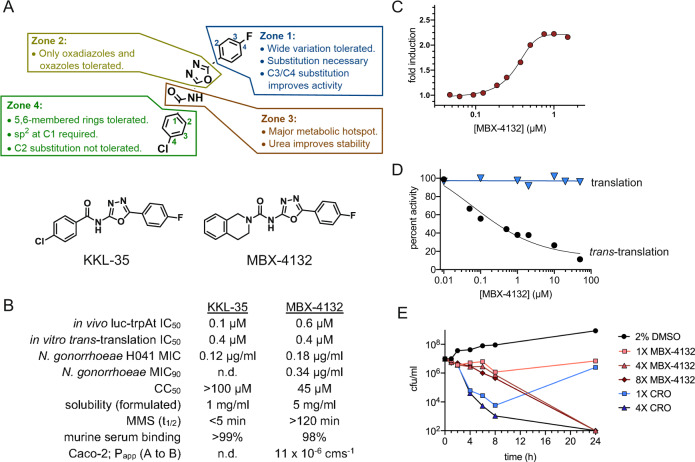
Optimized acylaminooxadiazoles inhibit *trans*-translation to kill *N. gonorrhoeae*. **A** Zones used to guide synthetic strategy with characteristics that govern activity are indicated, and the structures of KKL-35 and MBX-4132 are shown. **B** Properties of the initial hit, KKL-35, and optimized inhibitor MBX-4132 (CC_50_ – half-maximal cytotoxic concentration against HeLa cells; MMS – murine liver microsome stability). **C** Inhibition of *trans*-translation in *E. coli* cells was monitored using a non-stop luciferase reporter. The average of two biological repeats is shown. **D** Inhibition of *trans*-translation in vitro was assayed using an *E. coli* S12 extract to express a truncated, non-stop nano-luciferase gene in the presence of a mutant tmRNA that added the remainder of the nano-luciferase protein. *Trans*-translation activity resulted in luminescence, and addition of MBX-4132 inhibited the reaction (black). As a control, a full-length nano-luciferase gene was used to demonstrate that MBX-4132 does not inhibit translation (blue). The percentage of activity compared to activity in absence of MBX-4132 is shown from the average of at least two repeats. **E** Time-kill assays using *N. gonorrhoeae* show that MBX-4132 is bactericidal at ≥4X MIC. Ceftriaxone (CRO) was used as a control. Counts below the detection limit (100 cfu/ml) were plotted at 100 cfu/ml. Source data are provided as a Source Data file.

Evaluation of in vitro pharmacokinetic properties of the original hit, KKL-35, revealed that the amide bond is rapidly hydrolyzed in liver microsomes, making it unsuitable for use in animals (Fig. 1, Supplementary Table 1). To enable animal studies, >500 analogs of KKL-35 were designed and evaluated for potency, toxicity, and pharmacokinetic properties (Fig. 1, Supplementary Table 1). Compound potency, assessed by minimum inhibitory concentration (MIC) against *N. gonorrhoeae* and activity (EC_50_) in a *trans*-translation luciferase reporter assay^3^, was responsive to structural changes, consistent with specific binding of the molecule to the target (Fig. 1, Supplementary Table 1). Conceptually, the compound can be divided into 4 distinct zones (Fig. 1A). The central portion (Zones 2 and 3) played a critical role in activity, and the termini (Zones 1 and 4) tolerated changes that facilitated tuning physical properties and potency. A key finding of these experiments was that replacement of the Zone 3 amide with a urea dramatically improved metabolic stability without significantly decreasing potency (Fig. 1, Supplementary Table 1). MBX-4132, a uriedooxadiazole, exhibited excellent stability in both murine liver microsomes (t_1/2_ > 120 min.) and murine serum (>99.8% remaining after 1 h) (Fig. 1B, Supplementary Table 2). MBX-4132 also showed excellent permeability through Caco-2 cells, a predictive indicator of oral bioavailability (Fig. 1B, Supplementary Table 2). These data suggested that MBX-4132 could be dosed orally and was likely to be stable in animals. Like KKL-35, MBX-4132 inhibited *trans*-translation both in the *E. coli* luciferase reporter assay (Fig. 1B) and in an in vitro reconstituted assay (IC_50_ = 0.4 µM) but did not inhibit translation in vitro (Fig. 1D).

Like the parent acylaminooxadiazole compounds, MBX-4132 exhibited potent broad-spectrum antibiotic activity against Gram-positive species and many Gram-negative species, including *N. gonorrhoeae* (Fig. 1, Supplementary Tables 3 and 4). The prevalence of multiple-drug resistant (MDR) strains of *N. gonorrhoeae* has made infections increasingly difficult to treat^2^. MBX-4132 was highly effective against all tested clinical isolates of *N. gonorrhoeae*, including MDR strains (Fig. 1, Supplementary Table 3), indicating that prevalent resistance mechanisms are not active against MBX-4132. The frequency of spontaneous mutants resistant to MBX-4132 was <1.2 × 10^−9^, suggesting that emergence of resistance in the clinic is likely to be rare. Time-kill assays demonstrated that MBX-4132 was bactericidal against *N. gonorrhoeae* at concentrations ≥ 4X MIC (Fig. 1E).

In vitro analyses indicated that MBX-4132 is likely to have low toxicity in mammals. Assays for competitive binding with 45 mammalian receptors, inhibition of 7 cardiac ion channels, inhibition of the 5 major liver CYP450 enzymes, and an Ames assay for genotoxicity revealed only minor inhibition of two mammalian receptors, indicating that there is minimal off-target activity (Supplementary Tables 5 and 6). In addition, high concentrations of MBX-4132 had no effect on mitochondrial membrane polarity in human hepatocytes, although elevated levels of reactive oxygen species (ROS) were observed at cytotoxic compound concentrations (Supplementary Table 7). Likewise, MBX-4132 did not induce differential toxicity against HepG2 cells in the presence of either glucose or galactose, consistent with normal mitochondrial metabolism (Supplementary Table 7). Collectively, these data show that MBX-4132 is appropriate for animal model studies and potentially for human use.

### MBX-4132 clears multi-drug resistant *N. gonorrhoeae* from infected mice

The development of a single dose therapy for gonorrhea is clinically important as a means to ensure patient compliance and limit development of resistance^12,13^. As predicted by the in vitro pharmacokinetic assays, MBX-4132 was highly orally bioavailable in mice, exhibiting excellent plasma exposure (area under the curve; AUC), half-life (t_1/2_), and a low clearance rate (Supplementary Fig. 1). These data indicate the compound accumulates at high levels and provides prolonged exposure after a single oral dose. The mice exhibited no obvious adverse effects after a single dose of 100 mg/kg or repeat dosing at 10 mg/kg (BID, 7 d), demonstrating that the compound is tolerated well. Based on these results and the in vitro potency against *N. gonorrhoeae*, we investigated the efficacy of a single oral dose of MBX-4132 in a murine genital tract infection model^14–17^.

**Fig. 2:**
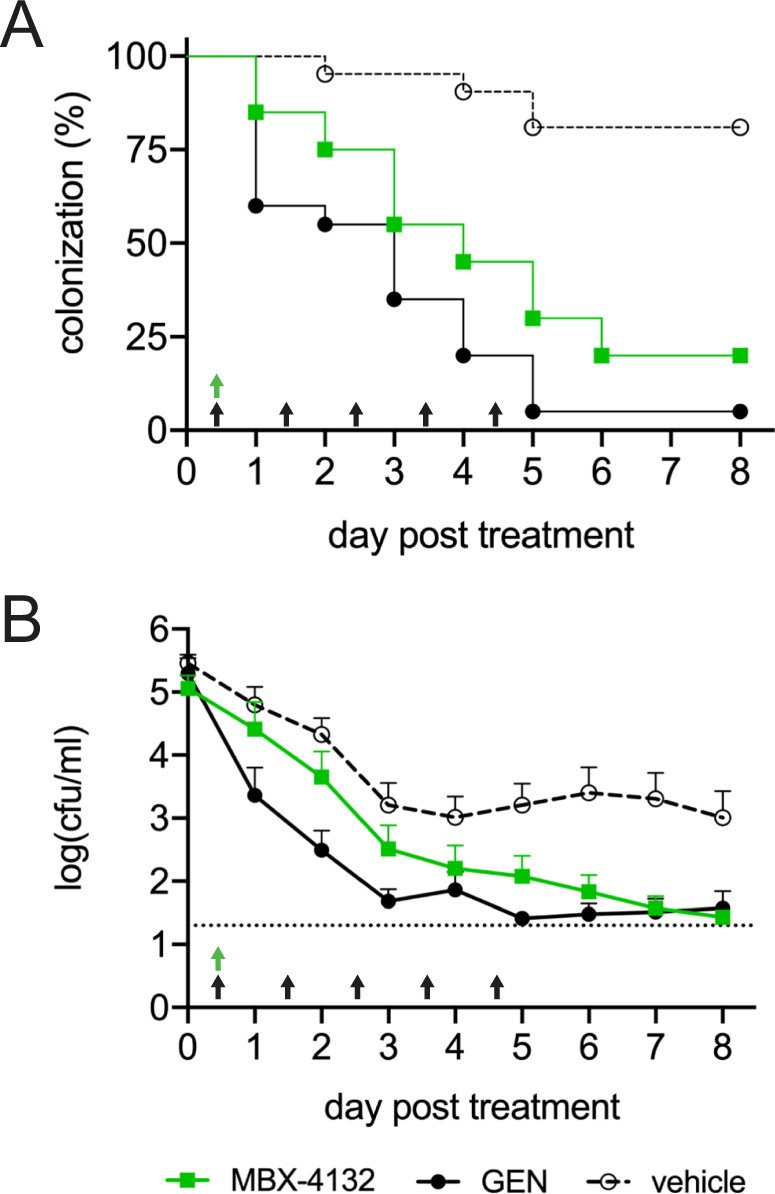
MBX-4132 clears infection by a multiple-antibiotic resistant *N. gonorrhoeae* strain in a murine infection model. Mice were infected with *N. gonorrhoeae* H041 for two days and treated with a single oral dose of 10 mg/kg MBX-4132 or vehicle on day 0 (green arrow), (*n* = 20 mice for MBX-4132 and *n* = 21 mice for vehicle). As a positive control, 48 mg/kg gentamicin (GEN) was administered by intraperitoneal injection beginning on day 0 (5QD, black arrows). **A** The percentage of infected mice over 8 days post-treatment. Mice that were culture-negative for at least 3 consecutive days were considered to have cleared infection. MBX-4132 and GEN significantly reduced the percent of infected mice compared to vehicle (Mantel-Cox, *p* < 0.0001). **B** Mean bacterial burden (cfu/ml) recovered daily following treatment on day 0. MBX-4132 and GEN significantly reduced the bacterial burden compared to vehicle (2-way ANOVA with Bonferroni for multiple comparisons, *p* < 0.0001). Limit of detection (20 cfu/ml) is denoted by the horizontal dashed line. Error bars indicate standard error of the mean. Source data are provided as a Source Data file.

To test in vivo efficacy, lower genital tract infection was established with the *N. gonorrhoeae* clinical isolate H041, an MDR strain that is resistant to at least 7 classes of antibiotics^18^. Infected mice were treated either with vehicle, a single oral dose of 10 mg/kg MBX-4132, or with daily intraperitoneal injection of 48 mg/kg gentamicin for 5 days (*n* = 20–21 mice/group). Efficacy was monitored by quantitative culture of *N. gonorrhoeae* from vaginal swabs, and clearance of infection from a mouse was defined as three consecutive days with no detectable *N. gonorrhoeae* (Fig. 2). Gentamicin was used as a positive control because it is one of the few antibiotics shown to be effective against H041 in vivo, even though it is not orally bioavailable and requires daily dosing^15^. As previously observed, daily intraperitoneal injection of gentamicin was effective against H041, clearing infection in 95% of treated mice (Fig. 2A). A single oral dose of MBX-4132 also showed significant efficacy, with 80% of mice completely cleared of infection by 6 days, and a dramatic reduction in bacterial load across all animals (Fig. 2B). These data are the first in vivo proof-of-concept that inhibition of *trans*-translation is a viable antibiotic strategy and are particularly noteworthy because they were achieved against an MDR strain of gonorrhea following a single oral dose.

### Acylaminooxadiazole inhibitors bind near the PTC of non-stop ribosomes

**Fig. 3:**
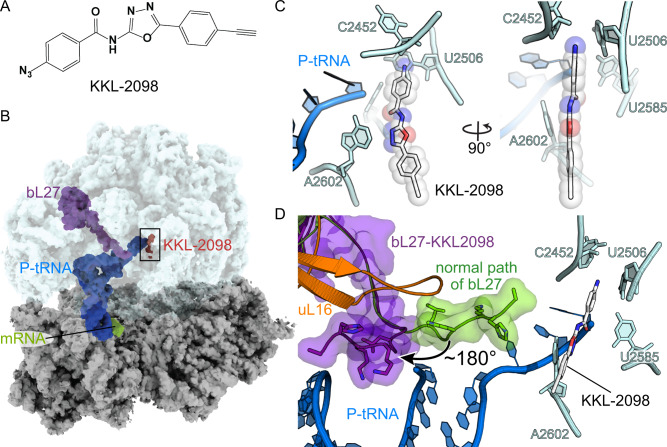
KKL-2098 binds near the peptidyl transferase center. **A** The chemical structure of KKL-2098. **B** The cryo-EM structure of the *E. coli* 70S non-stop ribosome with P-site tRNA, mRNA, ribosomal protein bL27 and KKL-2098 indicated. **C** KKL-2098 is positioned close to the peptidyl transferase center adjacent to 23S rRNA A2602, C2452, U2506, and U2585 and the CCA end of the P-site tRNA. **D** The N terminus of bL27 (purple) moves 180° to pack against ribosomal protein uL16 and the acceptor arm of the P-site tRNA. The normal position of bL27 in translating ribosomes is shown in green (PDB 6ENU).

To understand the mechanism of the acylaminooxadiazole antibiotics, we used cryogenic electron microscopy (cryo-EM) to determine the structure of KKL-2098 cross-linked to a non-stop ribosome (Fig. 3, Supplementary Fig. 2, Table 1). In previous in vivo experiments, KKL-2098 crosslinked to 23S rRNA in a small percentage of total ribosomes^11^. For structural studies, we affinity-purified non-stop ribosomes to increase the homogeneity of the sample and the occupancy of KKL-2098. Non-stop ribosomes were generated in *E. coli* by over-expression of an mRNA containing an RNase III cleavage site before the stop codon. Endogenous RNase III cuts this mRNA in vivo, and translation of the truncated mRNA produces non-stop ribosomes. The mRNA encodes a histidine tag followed by 49 amino acids, such that non-stop ribosomes will have the histidine tag from the nascent polypeptide extending from the exit tunnel, enabling affinity purification. KKL-2098 was added to the culture, UV radiation was used to stimulate cross-linking between KKL-2098 and the ribosomes, and the non-stop ribosomes were affinity-purified, vitrified, and visualized by cryo-EM. 474,382 particles were collected and classified to isolate the 70S ribosomes containing a P-site tRNA, and the structure was solved to 3.1 Å resolution (Supplementary Figs. 2–4).

**Table 1:** Cryo-EM data collection, refinement and validation statistics.

	70S • P-site tRNA • KKL-2098 (EMDB-20121) (PDB 6OM6)
Data collection and processing	
Magnification	59,000
Voltage (kV)	300
Electron exposure (e–/Å^2^)	58
Defocus range (μm)	1.5–3.5
Pixel size (Å)	1.191
Symmetry imposed	C1
Initial particle images (no.)	474,382
Final particle images (no.)	28,121
Map resolution (Å)	3.1
FSC threshold	0.143
Map resolution range (Å)	3.1
Refinement	
Initial model used (PDB code)	5MDV
Model resolution (Å)	3.1
FSC threshold	0.143
Model resolution (Å)	3.3
Map sharpening *B* factor (Å^2^)	88.04
Model composition	
Non-hydrogen atoms	144295
Protein residues	5715
Ligands	KKL: 1
*B* factors (Å^2^)	min/max/mean
Protein	96.44/652.55/167.47
Ligand	152.66
R.m.s. deviations	
Bond lengths (Å)	0.007
Bond angles (°)	0.897
Validation	
MolProbity score	1.73
Clashscore	6.66
Poor rotamers (%)	0.3
Ramachandran plot	
Favored (%)	94.7
Allowed (%)	4.22
Disallowed (%)	1.12

Inspection of the map revealed density consistent with KKL-2098 near residue C2452 in the PTC, a 23S rRNA nucleotide shown to crosslink with KKL-2098 in *E. coli*^3^. The resolution of KKL-2098 was not high enough to distinguish one end of the molecule from the other, but the molecule could be oriented with high confidence based on the following considerations. First, Zone 4 of KKL-2098 contains the moiety that crosslinks to C2452^3^, and in its current refined position this end of KKL-2098 is within 2.1 Å of C2452 (Fig. 3C and Supplementary Fig. 5). Second, Zone 1 of KKL-2098, which was highly tolerant of chemical changes (Fig. 1), is in a position where the alkyne does not make any molecular interactions with the ribosome, consistent with its chemical promiscuity. Third, in this orientation N3 of the oxadiazole, which allowed no substitutions (Fig. 1, Supplementary Table 1), is adjacent to A2602, positioned to make hydrogen-bonding or dipole interactions that require the 1,3,4 oxadiazole configuration (note that this oxadiazole configuration has a strong dipole moment^19^). Other molecular interactions between KKL-2098 and the ribosome include the amide oxygen in Zone 3 with the 3′ end of the terminal A76 from the P-site tRNA. Overall, these interactions explain why minor changes in Zones 2 and 3 dramatically reduced potency (Supplementary Table 1). The position of KKL-2098 would clash with tmRNA in the A site, partially overlapping with the phosphate of the terminal adenine (Supplementary Fig. 6), suggesting that the acylaminooxadiazoles may inhibit *trans*-translation in part either by preventing tmRNA-SmpB binding at the A site or by interfering with tmRNA-SmpB translocation from the A to the P site.

### The N terminus of bL27 is important for inhibition activity

Although no major rearrangements in the rRNA core of the PTC were observed, the N-terminal 7 residues of ribosomal protein bL27 (“b” indicates a bacterial specific ribosomal protein^20^) move ~180° from the PTC (Fig. 3D, Supplementary Movie 1). This position is >25 Å from its position in ribosomes containing a peptidyl-tRNA in the P site^21–23^ (Supplementary Fig. 7, Supplementary Movie 1). As is the case for many ribosomal proteins, the first 20 residues of bL27 form a long extension with no secondary structure and these residues are frequently not resolved in structures of 70S ribosomes lacking A-site tRNA. Cross-linking and biochemical studies show that the N terminus of bL27 extends to the 3ʹ end of the P-site tRNA and stabilizes product formation of the peptidyl transferase reaction^21,24–26^. Consistent with these data, the N terminus extends parallel to the 3ʹ end of the P-site tRNA in structures containing either peptidyl P-site tRNAs or aminoacylated A-site tRNAs^21–23^. In the KKL-2098-bound structure, the N terminus bends ~180° at Gly7 and packs against ribosomal protein uL16 (“u” indicates a universal ribosomal protein^20^) and the acceptor stem of the P-site tRNA. In this position bL27 would not be able to participate in the peptidyl transferase reaction when tmRNA is presented in the A site. The two distinct positions of bL27 suggest that its N terminus is mobile and its movement may be required for optimal *trans*-translation activity.

**Fig. 4:**
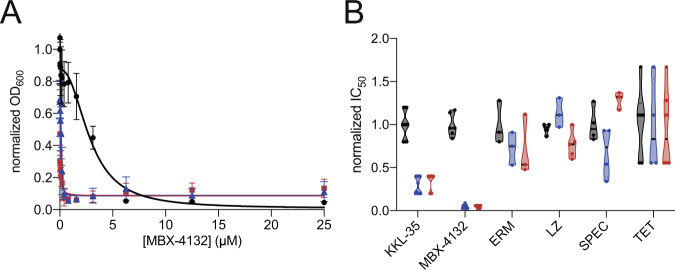
Truncation of bL27 causes hypersensitivity to acylaminooxadiazoles. **A** Growth of *E. coli ∆tolC* expressing full-length bL27 (black) or variants missing residues 2–4 (-3, blue) or 2–7 (-6, red) was monitored in broth microdilution experiments and the IC_50_ for MBX-4132 was determined. At least two technical replicates were performed for each biological replicate and the average of at least three biological replicates is shown with error bars indicating standard deviations. **B** Violin plot showing normalized mean IC_50_ values from at least 3 biological replicates of experiments as in (**A**) show that truncation of bL27 increases sensitivity to KKL-35 and MBX-4132 but has no effect on erythromycin (ERM), linezolid (LZ), spectinomycin (SPEC) or tetracycline (TET). Source data are provided as a Source Data file.

To test whether the position of bL27 plays a role in acylaminooxadiazole activity, we examined the effects of *E. coli* mutants that are truncated by 3 or 6 residues from the N terminus, preventing bL27 from reaching the PTC. These mutations decrease the rate of translation in vitro, but *E. coli* mutants containing only the truncated bL27 are viable, although they grow slowly^24^. Both mutants were hypersensitive to MBX-4132, but not to other antibiotics that target the ribosome, indicating that absence of bL27 from the PTC increases sensitivity to MBX-4132 (Fig. 4).

## Discussion

MBX-4132 can clear MDR *N. gonorrhoeae* from infected mice after a single oral dose, indicating that the compound is effective under an ideal clinical dosing regimen. The low toxicity against human cells, enzymes, and receptors suggests that MBX-4132 could be developed for clinical treatment of drug-resistant gonorrhea in humans. Taken together, the specific inhibition of *trans*-translation by acylaminooxadiazoles and significant in vivo efficacy against *N. gonorrhoeae* after a single oral dose, combined with the new chemical structure, support further development of these compounds as promising new antibiotics.

The mechanism of action for the acylaminooxadiazoles is important both for antibiotic development and for fundamental understanding of *trans*-translation. In particular, the mechanism for specifically inhibiting *trans*-translation but not translation is likely to reveal important differences between these reactions. In this context, the position of KKL-2098 on non-stop ribosomes was surprising. Binding of acylaminooxadiazoles could inhibit *trans*-translation by blocking tmRNA in the A site (Supplementary Fig. 6). However, given the similarities between the acceptor stems of tmRNA and tRNAs in the A site, why do molecules like MBX-4132 not inhibit translation in addition to *trans-*translation? One possible explanation is the difference in the positions of tmRNA and tRNA as they translocate through the ribosome. The acceptor stems of tmRNA and tRNA are similar after accommodation in the A site, but there are slight differences after peptidyl transfer when the molecules adopt hybrid A/P states (Supplementary Fig. 6)^27,28^. Moreover, unlike tRNA, tmRNA can adopt a hybrid A/P state without inducing rotation between the 50S and 30S ribosomal subunits, suggesting a substantial difference in the process of translocation (Supplementary Fig. 6)^27,28^. These differences between tmRNA and tRNA on the ribosome might allow MBX-4132 to specifically block tmRNA and thereby exclusively inhibit *trans*-translation.

**Fig. 5:**
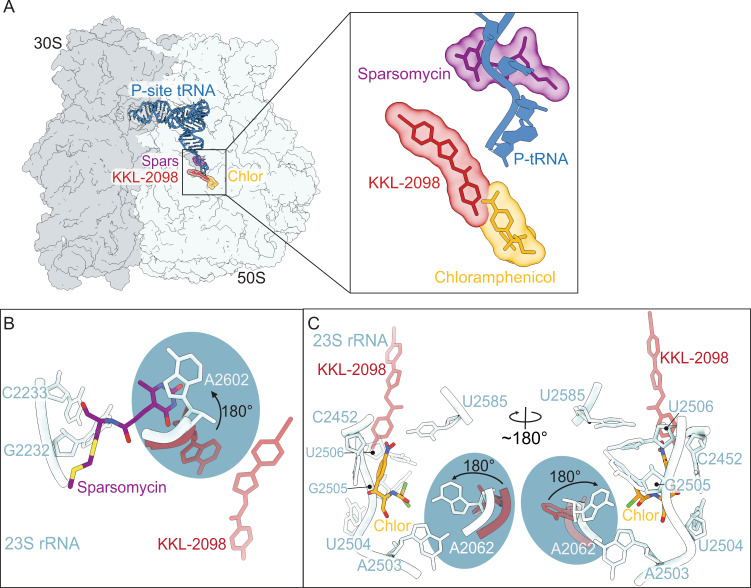
Comparison of the position of KKL-2098 to other PTC inhibitors. **A** Overview of the bacterial ribosome showing the position of sparsomycin (PDB 1NJM), chloramphenicol (PDB code 6ND5) and KKL-2098. **B** Inset view of the interactions of PTC nucleotides with sparsomycin. The nucleobase of 23S rRNA A2602 moves ~180° upon sparsomycin binding. KKL-2098 and A2602 in the absence of sparsomycin binding are shown in red. **C** Inset view of the interactions of PTC nucleotides with chloramphenicol. The nucleobase of 23S rRNA A2062 also moves ~180° upon chloramphenicol binding. KKL-2098 and A2062 in the absence of chloramphenicol binding are shown in red.

The overall binding site of KKL-2098 is distinct from antibiotics that inhibit peptidyl transferase activity, including sparsomycin and chloramphenicol (Fig. 5). Binding of sparsomycin and chloramphenicol alters the conformation of critical PTC nucleotides. Sparsomycin binds close to the P-site tRNA (on the side closest to the E site) but on the opposite side of the P-site tRNA from chloramphenicol and KKL-2098 (Fig. 5B)^29,30^. Sparsomycin causes the nucleobase of universally conserved 23S rRNA A2602 to rotate ~180°^30^. Chloramphenicol binds close to the CCA end of P-site tRNA and its binding causes the nucleobase of A2062 to rotate ~180°^29^. A2062 is also universally conserved and is important for sensing specific nascent chain residues in the exit tunnel that cause ribosome stalling^31,32^. In contrast, KKL-2098 binding is more distant from the PTC and does not induce changes in the positions of PTC nucleotides.

Conversely, binding of KKL-2098 alters the conformation of bL27 but antibiotics that target translation do not. The N terminus of bL27 is not resolved in most structures that do not contain an A-site ligand, including non-stop ribosomes without an inhibitor^22,33^, suggesting that the N terminus is mobile during translocation and accommodation of a new A-site ligand. In contrast, the presence of KKL-2098 in the non-stop ribosome stabilizes bL27 in the rotated conformation away from the PTC. If *trans*-translation specifically requires the N terminus of bL27 to be oriented toward the PTC, stabilization of the rotated conformation would be expected to inhibit *trans*-translation more than translation elongation. Alternatively, the acylaminooxadiazoles may specifically recognize subtle differences between non-stop ribosomes and translating ribosomes that are not observed in the structure described here. We note that the structural basis for the specificity of many ribosome-binding antibiotics has been difficult to discern^34^. For example, chloramphenicol binds in the A site near the PTC in a position that would appear to block any peptide-bond formation. However, many sequences are translated in the presence of chloramphenicol and inhibition depends on both the nascent polypeptide and the incoming amino acid^35,36^. Although further experiments will be required to test the models for specific inhibition of *trans*-translation, it is clear that the acylaminooxadiazoles exhibit a novel mechanism of antibacterial activity.

## Methods

### Chemical synthesis

All commercially obtained reagents and solvents were used as received. ^1^H and ^13^C NMR spectra were recorded on a Bruker 300 MHz instrument. Chemical shifts are given in ∂ values referenced to the internal standard tetramethylsilane^37^. LC/MS analyses were performed on a Thermo-Finnigan Surveyor LC unit connected to a Thermo LTQ Fleet MS unit. HPLC purification was performed on a Gilson Unipoint instrument equipped with a 00G-4252-P0-AX C18, 10 µm, 150 mm or 250 mm × 21.2 mm column from Phenomonex. Silica column purification was performed on Isco brand Combi-flash R_f_ liquid chromatography system using 50 µm silica Luknova SuperSep columns. Melting points were taken on EZ-Melt automated melting point apparatus (Stanford Research Systems, Inc.) in manual mode, and are uncorrected. Thin-layer chromatography was performed on silica gel GHLF plates from Analtech (Newark, DE), and the chromatograms were visualized under UV light at 254 nm. 5-(4-fluorophenyl)-1,3,4-oxadiazol-2-amine, 3-phenyl-1,2,4-oxadiazol-5-amine were purchased from Enamine (Kiev, Ukraine); 5-phenyl-1,2,4-oxadiazol-3-amine was purchased from Chembridge (San Diego, CA, USA); 5-cyclohexyl-1,3,4-oxadiazol-2-amine, 1,3,4-oxadiazol-2-amine were purchased from Life Chemicals (Niagara-on-the-Lake, ON, Canada); p-Toluenesulfonyl Chloride, 1,1’-Carbonyldiimidazole were purchased from Thermo Fisher Scientific (Acros Organics) (New Jersey, USA); 4-Chlorobenzoyl chloride was purchased from Thermo Fisher Scientific (Alfa Aesar) (New Jersey, USA); 2-amino-5-phenyl-1,3,4-oxadiazole amine, 4-Chlorobenzoic acid, Cyclohexanecarboxylic acid, 4-Chlorobenzaldehyde, Pyrrolidine, Piperidine, Hexamethyleneimine, Morpholine, Methyl Iodide, Sodium Triacetoxyborohydride, Diisopropylethyl amine, Triethylamine, and all solvents were purchased from Millipore-Sigma (St. Louis, MO, USA); 1,2,3,4-tetrahydro-isoquinoline and 4-chlorobenzene sulfonyl chloride were purchased from Combi-Blocks (San Diego, CA, USA); 4-methyl-1,2,3,6-tetrahydropyridine hydrochloride was purchased from Pharmablock (Hatfield, PA, USA); HATU was purchased from GenScript (Piscataway, NJ, USA).

#### General method A

Heteroaryl amine (1.1eq) was dissolved in NMP (0.29 M) and triethylamine (1.2 eq) stirred for 10 min at room temperature; to this solution, acid chloride (1 eq) was added dropwise via pipette. The reaction mixture was allowed to continue stirring at room temperature for 16–20 h. If the reaction did not reach completion, the mixture was warmed to 50 °C for an additional 16–20 h. Upon completion, reaction mixture was added to water, and the resulting precipitate was filtered and dried. Crude precipitate purified with HPLC (5–95% MeCN/H_2_O + 0.1% TFA).

#### General method B

Carboxylic acid (1.1 eq) and HATU/HBTU (1.2 eq) were dissolved in NMP (0.28 M), to which N,N-diisopropylethylamine (1.2 eq) and heteroaryl amine (1 eq) were added. The reaction was stirred at room temperature for 16–20 h. If the reaction did not reach completion, the mixture was warmed to 50 °C for an additional 16–20 h. Upon completion, reaction mixture was added to water, and the resulting precipitate was filtered and dried. Crude precipitate purified with HPLC (5–95% MeCN/H_2_O + 0.1% TFA).

#### General method C

To oxadiazole amine (eq) and 1,1’-Carbonyldiimidazole (1 eq) in N-methyl imidazole (0.56 M) was added 3 Å molecular sieves (1.6 mm pellets: ~4 pellets/mmol). Reaction was stirred at room temperature for 3–16 h. Then, the second amine (1.1 eq) was added to the reaction and continued stirring at room temperature for 2–18 h. Upon completion, reaction mixture was added to water, and the resulting precipitate was filtered and dried. Crude precipitate was purified with HPLC (5–95% MeCN/H_2_O + 0.1% TFA).

#### KKL-35/MBX-3535

Prepared according to General Method A to provide material consistent with prior reports^38^. 15.0 mg (7%); mottled tan powder; ^1^H NMR (DMSO): 12.35 (br, 1H), 8.08–8.01 (m, 4H), 7.65 (d, 2H), 7.47 (t, 2H); LC/MS: 318.0 (M + 1); mp: >240 °C (decomp.); R_f_: 0.25 (50% EtOAc/hexanes).

#### MBX-4083

Prepared according to General Method A. 26.0 mg (14%); off-white solid; ^1^H NMR (DMSO): 12.75 (br s, 1H), 8.07–7.99 (m, 4H), 7.67–7.58 (m, 5H); LC/MS: 300.0 (M + 1); mp: 182–188 °C; R_f_: 0.64 (50% EtOAc/hexanes).

#### MBX-3943

Prepared according to General Method A. 12.0 mg (6%); white solid; ^1^H NMR (DMSO): 11.77–11.68 (m, 1H), 8.14–7.91 (m, 4H), 7.76–7.53 (m, 5H); LC/MS: 300.2 (M + 1); mp: >102 °C (slow); R_f_: 0.65 (50% EtOAc/hexanes).

#### MBX-3910

Prepared according to General Method A to provide material consistent with prior reports^39^. 30.0 mg (16%); white solid; ^1^H NMR (DMSO): 12.26 (br, 1H), 8.08–7.96 (m, 4H), 7.67–7.62 (m, 5H); LC/MS: 300.2 (M + 1); mp: 250–259 °C; R_f_: 0.34 (50% EtOAc/hexanes).

#### MBX-4370

Prepared according to General Method A to provide material consistent with prior reports^38^. 26.0 mg (28%); White solid; ^1^H NMR (DMSO): 11.95 (bs, 1H), 8.02–8.00 (m, 2H), 7.64–7.61 (m, 2H), 2.94 (m, 1H), 2.02–1.98 (m, 2H), 1.77–1.41 (m, 8H); LC/MS: 306.2 (M + 1); mp: 204–206 °C; R_f_: 0.72 (5% MeOH/DCM).

#### MBX-4367

Prepared according to General Method A. 17.0 mg (13%); white solid; ^1^H NMR (DMSO): 12.12 (bs, 1H), 9.10 (s, 1H), 8.06–7.98 (m, 2H), 7.65–7.59 (m, 2H); LC/MS: 224.0 (M + 1); mp: 212–214 °C; R_f_: 0.32 (5% MeOH/DCM).

#### MBX-C4227

Material was purchased and tested as received from Life Chemicals, Inc.

#### MBX-3709

Prepared according to General Method A. 150 mg (97%); light brown solid; ^1^H NMR (DMSO): 11.66 (s, 1H), 7.99–7.95 (m, 2H), 7.47–7.41 (m, 2H), 1.87–1.66 (m, 6H), 1.41–1.22 (m, 6H); LC/MS: 290.0 (M + 1); mp: >205 °C (slow); R_f_: 0.73 (3.75:46.25:50 MeOH/EtOAc/DCM).

#### MBX-3776

Prepared in a manner analogous to that previously described^40^, by dissolving (E/Z)-N-(4-chlorobenzylidene)-5-(4-fluorophenyl)-1,3,4-oxadiazol-2-amine (0.331 mmol, 1.0 eq), and NaHB(OAc)_3_ (0.430 mmol, 1.3 eq) in dichloromethane (1 ml), stirred at room temperature for 18 h. Adsorbed material onto Celite and isolated product from column chromatography eluted with linear gradient of 0–50% EtOAc in Hexanes. 27.0 mg (27%); White solid; ^1^H NMR (DMSO): 8.39 (t, 1H), 7.87–7.82 (m, 2H), 7.41–7.34 (m, 6H), 4.43 (d, 2H); LC/MS: 304.1 (M + 1); mp: 165–167 °C; R_f_: 0.42 (50% EtOAc/hexanes).

#### MBX-4076

Prepared by dissolving 4-chloro-N-(5-(4-fluorophenyl)-1,3,4-oxadiazol-2-yl)benzamide (MBX-3535, 0.220 mmol, 1.0 eq) in DMF (2 ml), to which K_2_CO_3_ (0.264 mmol, 1.1 eq) was added and the mixture was stirred at room temperature for 2 h. Then methyl iodide (0.220 mmol, 1.0 eq) was added and continued to stir at room temperature for an additional 65 h. The reaction was diluted with water (~30 ml), solid precipitate was filtered and dried under high vacuum. Product was purified by HPLC (20–100% MeCN/H_2_O + 0.1% TFA) and freeze dried to solid. 13.0 mg (18%); White solid; ^1^H NMR (CDCl_3_): 8.21 (d, 2H), 8.01–7.97 (m, 2H), 7.40 (d, 2H), 7.22 (t, 2H), 3.74 (s, 3H); LC/MS: 332.2 (M + 1); mp: >148 °C (slow); R_f_: 0.66 (50% EtOAc/hexanes).

#### MBX-4063

Prepared according to modified General Method A; used solvent mixture of Pyridine/Dichloromethane (1/1 mixture) instead of NMP and trietheylamine. 33.0 mg (18%); white solid; ^1^H NMR (CDCl_3_): 7.96–7.86 (m, 4H), 7.34–7.16 (m, 4H + CHCl3), 2.42 (s, 3H); LC/MS: 334.1 (M + 1); mp: 218–221 °C; R_f_: 0.14 (50% EtOAc/hexanes).

#### MBX-4346

Prepared according to General Method C. 15.0 mg (20%); light yellow solid; ^1^H NMR (CDCl_3_): 7.98–7.95 (m, 2H), 7.20–7.14 (m, 2H), 3.57 (m, 4H), 1.96 (m, 4H); LC/MS: 277.1 (M + 1); mp: 197–199 °C; R_f_: 0.94 (10% MeOH/DCM).

#### MBX-4699

Prepared according to General Method C. 13.0 mg (9%); white solid; ^1^H NMR (CDCl_3_): 7.97–7.92 (m, 2H), 7.21–7.15 (m, 2H), 3.65 (m, 4H), 1.62 (m, 6H); LC/MS: 291.5 (M + 1); mp: 200–203 °C; R_f_: 0.64 (10% MeOH/DCM).

#### MBX-4700

Prepared according to General Method C. 15.0 mg (33%); white solid; ^1^H NMR (CDCl_3_ + MeOD): 7.92–7.88 (m, 2H), 7.13–7.07 (m, 2H), 3.48 (m, 4H), 1.69 (m, 4H), 1.52–1.50 (m, 4H); LC/MS: 305.7 (M + 1); mp: 191–194 °C; R_f_: 0.63 (10% MeOH/DCM).

#### MBX-4697

Prepared according to General Method C. 8.9 mg (15%); white solid; ^1^H NMR (CDCl_3_ + MeOD): 7.93–7.89 (m, 2H), 7.18–7.12 (m, 2H), 3.66 (m, 8H); LC/MS: 293.9 (M + 1); mp: 211–216 °C; R_f_: 0.55 (10% MeOH/DCM).

#### MBX-4366

Prepared according to General Method C. 19.0 mg (23%); white solid; ^1^H NMR (CDCl_3_): 7.98–7.93 (m, 2H), 7.21–7.16 (t, 2H), 5.41 (m, 1H), 4.11 (m, 2H), 3.78 (m, 2H), 2.11 (m, 2H), 1.73 (s, 3H); LC/MS: 303.0 (M + 1); mp: 180–181 °C; R_f_: 0.74 (5% MeOH/DCM).

#### MBX-4132

Prepared according to General Method C; dried precipitate solids were triturated in MeOH (~50 mL) yielded pure product. 375 mg (40%); white solid; ^1^H NMR (DMSO): 7.98–7.94 (m, 2H), 7.46–7.40 (m, 2H), 7.19 (s, 4H), 4.69 (s, 2H), 3.75 (t, 2H), 2.85 (t, 2H); LC/MS: 339.1 (M + 1); mp: >190 °C (slow); R_f_: 0.51 (50% EtOAc/hexanes).

### Bacterial strains and growth conditions

Bacterial strains, plasmids, and synthetic sequences are shown in Supplementary Table 8. *E. coli* strains expressing bL27 (“b” indicates bacterial specific ribosomal proteins^20^) were constructed by transducing a *tolC::cat* allele into IW312 strains using a P1 *vir* phage. These strains were grown in LB medium containing 1 mM IPTG. To quantify the in vitro antibacterial activity of acylaminooxadiazole analogs against various bacterial strains, the minimum inhibitory concentration (MIC) was measured using microbroth dilution assays as described in the CLSI guidelines (M7-A7)^41^, except that liquid G77L medium^42^ was used for *Neisseria gonorrhoeae* MIC assays. Each assay comprised three technical replicates, and each assay was repeated at least once. The reported MIC is the geometric mean of at least 5 technical replicates. H041(STM^R^) is a streptomycin-resistant derivative of the multi-drug resistant (MDR), ceftriaxone-resistant strain H041 (Ohnishi) and was cultured as previously described^15,18,43^.

### Time-kill assays

The time kill assay was performed essentially as described^44^ with the following modifications for *N. gonorrhoeae*. The bacterial inoculum for the assays was prepared by suspending colonies of *N. gonorrhoeae* ATCC 49226 grown on a chocolate agar plate for >24 h (37 °C with 5% CO_2_) in G77L medium. The cell suspension was adjusted to an OD_600_ of 0.1 and was diluted 1:10 in G77L media (final cell density ~1 × 10^7^ cells/ml) containing various concentrations of MBX-4132. The resulting cultures were incubated at 37 °C (5% CO_2_), and viability was monitored over 24 h by removing samples at various time points, making serial 10-fold dilutions in G77L, and spotting 5 µl of each dilution onto the surface of a chocolate agar plate in triplicate. Colonies were counted after the plates were incubated at 37 °C (5% CO_2_) for 18–18 h, colony forming units (cfu) per ml were calculated, and the average and standard deviation for the three replicates was determined. The lower limit of detection of this assay was determined to be 100–200 cfu/ml. This experiment was repeated three times, and the results of a representative experiment are shown.

### In vitro *trans*-translation and translation assays

The in vitro *trans*-translation assay is designed to produce the first 157 amino acids of nano-luciferase from a non-stop mRNA (nanoluc-ns) and add the C-terminal peptide AAVSGWRLFKKIS via a mutant version of *E. coli* tmRNA (tmRNA-nl) to reconstitute an active nano-luciferase^45^. In this assay, the tagged nano-luciferase gene has >30,000-fold increase in activity over the peptide made in the absence of tmRNA-nl.

S12 lysates were made according to the procedure of Kim, et al.^46^. Briefly, a culture of *E. coli* BL21 (DE3) cells containing a pET28 plasmid carrying the *E. coli* T7 polymerase gene was grown at 37 °C to an OD_600_ = 0.8, induced with 1 mM IPTG and grown for an additional 3 h. Cells were harvested by centrifugation at 20,000 × *g* for 10 min at 4 °C and the pellet was resuspended in buffer A (20 mM Tris-acetate (pH 8.2), 14 mM Mg(OAc)_2_, 60 mM potassium glutamate, 1 mM DTT). Cells were lysed by sonication, the lysate was clarified by centrifugation at 12,000 × *g* for 10 min, and the supernatant was stored at −80 °C.

*E. coli* SmpB was purified as previously described^3^. tmRNA-nl, a variant of *E. coli* tmRNA encoding the final 11 residues of nano-luciferase, was transcribed from the tmRNA-nl synthetic DNA sequence in vitro, purified, and folded as previously described for wild-type *E. coli* tmRNA^3^. The nanoluc-stop template DNA was prepared as previously described^3^ by PCR amplification from pMC1 using T7 universal and nanoluc-stop primers. The nanoluc-ns was prepared in a similar manner using pMC1 with T7 universal and nanoluc-ns primers.

For in vitro *trans*-translation, tmRNA-nl and SmpB were premixed and stored on ice. S12 lysate (2 μl), freshly made polymix buffer (2 μl)^47^ (final reaction concentrations 5 mM Hepes pH 7.6, 5 mM NH_4_Cl, 0.5 mM CaCl_2_, 1.5 mm MgCl_2_, 1 mM DTT, 8 mm putrescene, 2 mm ATP, 2 mM GTP, 1 mM CTP, 1 mM UTP, 0.3 mM each amino acid, 3 mg/ml *E. coli* tRNAs), nanoluc-ns template (0.5 μl; 60 ng) and 2 µl water were mixed and incubated at 37 °C for 5 min. This solution was dispensed to tubes containing 0.5 μl of different concentrations of an inhibitor prepared in 75% acetonitrile, 25% water, and incubated at 37 °C for 5 min. The tmRNA-nl/SmpB mixture (0.5 μl each, 2 μM final) was added to each tube and the samples were incubated at 37 °C for 1.5 h. NanoLuc substrate (Promega) was prepared according to the manufacturer’s instructions, one volume of this substrate solution was added to each sample tube, and the reactions transferred to a white 96-well plate. Luminescence readings were obtained using the SpectraMax i3 microplate reader. Data analysis was performed using GraphPad Prism 8.

### Frequency of resistance

First, MIC values were determined for MBX-4132 using the reference agar dilution method^41,48^. *N. gonorrhoeae* strain 49226 (ATCC) was suspended to the equivalent of a 5 McFarland standard in G77L broth and then diluted to generate the final inoculum (1.5 × 10^5^). The bacterial cell suspension was then transferred to wells in a stainless-steel replicator block which was used to inoculate the test plates. After the inoculum had dried, all plates were incubated at 35 °C in 5% CO_2_. The MIC was read post-incubation per CLSI guidelines^41,48^. To determine the frequency of resistance, stock solutions of MBX-4132 were prepared at 100X the final test concentrations of 4× and 8× the predetermined MIC value. A 0.5 mL aliquot of the 100X stock was mixed with 49.5 mL of molten GC Medium agar/1% IsoVitaleX to produce an agar/drug mixture that was either 4- or 8-fold the MIC and dispensed into sterile 150 × 15 mm plates (VWR) at a volume of 50 mL per plate. A dense cell suspension equivalent to 5 McFarland was prepared using bacterial growth from 48 h chocolate agar plates of *N. gonorrhoeae* (ATCC 49226). The viable count of each suspension was determined by plating serial ten-fold dilutions onto GC Medium agar/1% IsoVitaleX in duplicate. A 0.25 ml aliquot of inoculum was spread onto the surface of duplicate 150 × 15 mm test plates. After allowing the inoculum to dry on the surface of the plate, the plates were inverted and incubated at 35 °C (with 5% CO_2_) for 48 h. Colony counts were determined manually and the spontaneous mutation frequency was calculated using the following equation:

#### Average number of colonies from selection plates/Total number of cells inoculated

If there were no colonies on the antibiotic selection plates, the spontaneous mutation frequency was calculated as 1/inoculum to indicate that the spontaneous mutation frequency was less than the limit of detection (one cfu).

### Mammalian cell cytotoxicity (CC_50_)

The half maximal cytotoxic concentration (CC_50_) of each compound against HeLa cells (ATCC CCL-2) was measured as previously described^49^. Each assay comprised three technical replicates, and each assay was repeated at least once. The mean values for each biological replicate were averaged, and the CC_50_ was determined using a 4-parameter nonlinear curve fitting algorithm (GraphPad Prism). The average of the CC_50_s from two biological replicates was calculated and reported.

### Liver microsome stability

To examine potential for first-pass metabolism of analogs in the liver, the stability of analogs in the presence of liver microsome preparations (Eurofins Discovery for human, dog and rat; Xenotech for mouse) was measured using the method of Kuhnz, et al.^50^. for murine studies and Oback for the dog, human and rat studies^51^. The amount of parent compound remaining after incubation with microsomes in the presence of NADPH over a 30 min time range was measured using a reverse-phase liquid chromatography/mass spectroscopy method that was customized for each compound. Half-lives were calculated using linear regression analysis of several time points.

### Caco-2 permeability

To evaluate the potential for oral bioavailability, the ability of prioritized compounds to permeate a monolayer of Caco-2 (ATCC HTB-37) intestinal epithelial cells was determined as described^52^. Caco-2 permeability values (P_app_) > 1 × 10^−6^ cm/sec are predictive of oral bioavailability. The observation that P_app A→B_ > P_app B→A_ indicates that efflux from the basolateral compartment does not occur.

### Serum protein binding

Serum protein binding was determined using an equilibrium dialysis method as described^53^. The amount of compound in each chamber (buffer and serum) was measured using methods for reverse-phase liquid chromatography/mass spectroscopy methods that were customized for each compound.

### Aqueous solubility

The maximum aqueous solubility of each compound was determined using a nephelometric method as described^54^. Each assay comprised three technical replicates, and each assay was repeated at least once. The reported solubility is the average of at least 5 technical replicates.

### Cell-based non-stop luciferase reporter assay

To verify that MBX-4132 retains activity as an inhibitor of *trans*-translation, we measured its dose-dependent activity against the non-stop luciferase reporter assay strain E. coli SB75 *ΔtolC::kan* (pluc-trpAt) essentially as described^3^, with modifications. Briefly, serial 1.5-fold dilutions of MBX-4132 in DMSO were transferred to 96-well assay plates (Costar 3195), followed by the addition of 50 µl of an overnight culture of SB75 ΔtolC::kan (pluc-trpAt) that had been diluted to a final OD_600_ of 0.4 with LB media supplemented with 100 µg ampicillin/ml and 1 mM IPTG. The final concentrations of MBX-4132 ranged from 0.05 to 1.5 µM, and the final concentration of DMSO was 2%. The assay plates were incubated at room temperature for 2 h, and 50 µl of BrightGlo (Promega) bioluminescence reagent was added to each well. After 10 min incubation, bioluminescence intensity was measured using an Envision multi-label plate reader (Perkin Elmer). Each assay comprised three technical replicates, and the experiment was repeated three times. The fold induction for each MBX-4132 treated sample as compared to the DMSO-only sample was calculated for each technical replicate, and the average and standard deviation of the three technical was calculated. The IC_50_ was determined using a 4-parameter nonlinear curve fitting algorithm (GraphPad Prism).

### CYP450 inhibition, receptor panel profiling, and cardiac ion channel profiling

Several in vitro selectivity assays were performed at Eurofins Discovery Services using established methods and controls that behaved as expected (Supplementary Tables 5 and 6). For CYP450 inhibition assays, activity of MBX-4132 was tested at 5 concentrations from 30 nM to 100 μM; no inhibitory activity >50% was observed at any concentration. For receptor panel profiling, MBX-4132 was evaluated at 10 μM and activity of >50% (agonist or antagonist; note that negative % inhibition indicates agonism) was scored as active.

### Ames assay

The Ames assay was performed at SRI Biosciences, following the standard protocols established there^55–57^. In brief, samples were evaluated for their ability to induce genetic damage using the plate incorporation method with *Salmonella typhimurium* strains TA98 and TA100 with and without a metabolic activation mixture containing 10% Aroclar-1254-induced rat-liver microsomes (S9). MBX-4132 was tested from a 5 mg/mL DMSO stock solution (the solubility limit), which provided plate concentrations up to 500 µg/plate, with serial dilutions accessing doses as low as 5 µg/plate. MBX-4132 precipitated at the two highest dose levels (500 and 100 µg/plate), but at 50 µg/plate, no precipitation was observed. Precipitation did not interfere with colony counts or analysis. Test articles were considered mutagenic when the mean number of revertant colonies increased in a dose-dependent manner. Some cytotoxicity was observed, but sufficient colonies were formed to allow analysis. As shown in Supplementary Table 6, MBX-4132 exhibited no significant deviation in revertant colonies from the DMSO control at any concentration tested. Data for 2-nitrofluorene and sodium azide (positive controls) and DMSO (negative control) were included for reference.

### Mitochondrial toxicity assays

Multiplexed cytotoxicity assay — Human primary hepatocytes were grown on collagen I coated optical plates, cultured in hepatocytes culture media in a humidified 5% CO_2_ atmosphere at 37 °C. Cells were incubated in the presence of MBX-4132 at 10 concentrations starting a 100 µM and serially diluted 3.16-fold for 24 h at 37 °C, incubated for 30 min with multiplexed fluorescent dyes (Hoescht, 6-carboxy-2’,7’-dichlorodihydrofluorescein diazetat, and TMRE) and imaged to allow visualization of nuclei, reactive oxygen species generation, and mitochondrial oxidation. A >3.5-fold induction of ROS was considered consistent with formation of ROS and a >2-fold change in TMRE signal indicated an increase or decrease in mitochondrial membrane potential^58^.

Mitochondrial toxicity assay — HepG2 cells (ATCC) were seeded and cultured in media containing either glucose or galactose overnight. Test compounds were added at 8 concentrations (threefold serial dilution from 100 µM to 30 nM) and incubated with the cells for 24 h. Cell viability was measured by the alamarBlue method. The data in Supplementary Table 7 are inconsistent with MBX-4132 disrupting mitochondrial metabolic processes^59^. Assays were performed at Eurofins Cerep Panlabs.

### Pharmacokinetic analyses in mice

#### For PO suspension dosing (Performed at Neosome LLC)

Female CD-1 mice as above were fasted for 2 h prior, and 4 h after dosing. MBX-4132 was administered at 10 mL/kg via oral gavage with a 1.0, 2.5, or 10.0 mg/ml suspension in vehicle A (5% DMSO, 5% Cremophor EL^®^, 0.45% hydroxypropylmethylcellulose, 0.45% alginic acid, 22.5% hydroxy-betacyclodextrin). At 0.25, 0.5, 1, 2, 4, 8, and 24 h post dose, 3 mice from each group were euthanized by CO_2_ inhalation, blood was collected by cardiac puncture into K_2_EDTA collection tubes, and protein was precipitated and analysed by LC/MS-MS for plasma concentrations of MBX-4132. Data were analysed using WinNonLin.

#### For IV and SC dosing (performed at Charles River Labs)

MBX-4132 was administered at 6 ml/kg via direct tail vein puncture (IV, slow push) or subcutaneously to the intrascapular region (SC) in a 2 mg/ml 10% DMSO/80% PEG-400/10% water formulation. At 0.083, 0.5, 1, 4, 8 and 24 h post dose, blood was collected from 3 mice/group by tail vein or facial bleed into K_2_EDTA collection tubes, and protein was precipitated and analysed by LC/MS-MS for plasma concentrations of MBX-4132. Sampling was performed serially, with each mouse contributing sample at all time points. Data were analysed using WinNonLin.

### Murine tolerability studies

Single dose tolerability studies — Female CD-1 mice were fasted for 2 h prior, and 4 h after dosing. MBX-4132 was administered at 10 ml/kg via oral gavage with a 0.0 (vehicle control), 1.0, 2.5, or 10.0 mg/ml suspension in vehicle A. Mice were observed at 0.083, 0.25, 0.5, 1, 2, 5, 8, and 24 h post dosing, and any abnormal observations (behavior, agility, coat condition and appearance, color of urine, quality of feces, etc.) were noted. No abnormal observations of any dosing group were made during the 24 h course of this study. 4–6 weeks-old CD-1 female mice were received from Charles River laboratories and acclimated for at least 5 days prior to the start of the study. Apart from specific instances of fasting described in the experimental, the mice had free access to food and water. The animals were housed 3–6 per cage in a temperature and humidity-controlled room with a 12 h light cycle. All procedures described are in compliance with the Animal Welfare Act, the Guide for the Care and Use of Laboratory Animals, and the Office of Laboratory Animal Welfare. These studies conform to the NeoSome IACUC policies and Operational Guidelines as approved by the IACUC membership, NeoSome Safety Officers, or Attending Veterinarian. All testing was performed at Neosome LLC.

Multidose tolerability studies — In preparation for this study, an abbreviated version of the above murine PK study using the same formulation (A) examined fasted mice vs. fed mice was performed, with plasma samples taken at 1 and 4 h post dosing and analyses as described in the PK section. No significant variation in exposure was observed at either timepoint.

Female CD-1 mice were given free access to food and water. MBX-4132 was administered to two groups of 3 mice for 7 d either QD (with compound) or BID, with an additional two groups of mice given only vehicle at 10 ml/kg via oral gavage with a 1.0 mg/ml suspension in vehicle A. Mice were observed visually twice daily for 10 d (3 d post final dose), additionally, mice were weighed once daily. Any abnormal observations (behavior, agility, weight, coat condition and appearance, color of urine, quality of feces, etc.) were noted. No abnormal observations of any dosing group were made during the 24 h course of this study. One mouse in the vehicle only group did exhibit a slight weight loss but had recovered weight by the end of the study.

### In vivo efficacy testing in the gonorrhea mouse model

Groups of female BALB/cAnNCr mice (Charles River Laboratories) (6–7 weeks old) were treated with 17β-estradiol and antibiotics (streptomycin and trimethoprim) to increase susceptibility to long-term *N. gonorrhoeae* infection as described^15^. Mice were inoculated vaginally with *N. gonorrhoeae* strain H041 (10^4^ cfu/mouse) two days after estradiol pellet implantation and vaginal swabs were cultured for 2 days post-bacterial inoculation to confirm infection. On the afternoon of the second culture day (day 0), mice were given MBX-4132, GEN or the vehicle (*n* = 20–21 mice/group). Doses of MBX-4132 were prepared fresh in vehicle at the time of treatment and administered as a single oral dose (dose volume 10 ml/kg). The positive control GEN (48 mg/kg) was prepared and administered intraperitoneally as 5 daily doses as previously described^15^. Vaginal swabs were collected on 8 consecutive days following treatment and quantitatively cultured for *N. gonorrhoeae* to assess efficacy. The data are expressed as CFU/ml of vaginal swab suspension. Clearance was shown by Kaplan-Meier curves with log-rank (Mantel-Cox) statistical analysis. The average cfu/ml over time was compared by 2-way ANOVA with Bonferroni post-hoc analysis. Statistics were performed in GraphPad Prism Software. Mice were housed in Allentown cage units with 2–5 animals per cage at 20–26 °C with relative humidity 30–70% and a minimum of 10 air changes/h and 12 h light/12 h dark cycle. At the study endpoint (10 days post-inoculation), mice were euthanized using compressed CO_2_ gas in a CO_2_ gas chamber in the Laboratory Animal Medicine Facility. All animal experiments were conducted at the Uniformed Services University of the Health Sciences, a facility fully accredited by the Association for the Assessment and Accreditation of Laboratory Animal Care, under a protocol that was approved by the university’s Institutional Animal Care and Use Committee.

### Purification of stalled ribosome complexes

To construct pET28-H10arfArnc, a DNA cassette encoding 10 histidine residues, the M2 epitope, 3 glycine residues, and 20 arbitrary codons followed by 71 base pairs from the 3ʹ end of the *E. coli arfA* gene was synthesized and assembled into pET28 that had been digested with NcoI and HindIII^60^. The sequence from *arfA* contains the RNase III cleavage site^61,62^. Translation of the cleaved mRNA produces a 59 amino acid peptide with 10 histidines at the N terminus.

*E. coli* 70S ribosomes were purified as described previously^63^. *E. coli* BL21(DE3) pET28-H10arfArnc cells were grown to an *A*_600_ of ∼0.5 in Luria broth (LB) medium at 37 °C and induced with 1 μM IPTG and 1 μM KKL-2098 (synthesized as previously described^11^) and continued to grow for an additional hour at 37 °C then cooled on ice for 20 min. All centrifugation steps were performed at 4 °C. Cells were pelleted by centrifugation and washed with buffer 1 (10 mM HEPES-KOH, pH 7.6, 10 mM MgCl_2_, 1 M NH_4_Cl, 6 mM β-mercaptoethanol (β-Me)) twice and then resuspended in buffer 2 (10 mM HEPES-KOH, pH 7.6, 10 mM MgCl_2_, 100 mM NH_4_Cl, 6 mM β-Me). The cells were crosslinked under ultraviolet light (254 nm) for 10 min, then lysed using an EmulsiFlex-C5 high-pressure homogenizer (Avestin). Cell debris was removed by centrifuging at 13,000 × *g* for 15 min. The lysate was further centrifuged at 27,000 × *g* for 30 min to obtain the S30 fraction. Ribosomes were pelleted by centrifuging at 42,000 × *g* for 17 h. The pellets were resuspended in buffer 2 and bound to a 1 mL IMAC gravity column, washed with 10 column volumes of Buffer 2 and eluted with Buffer 2 supplemented with 500 μM imidazole. Ribosomes were further purified over a 10–40% sucrose gradient in buffer 2 at 70,000 × *g* for 12 h. 70S ribosomes were separated from polysomes and subunits using a Brandel gradient fractionator. The 70S fractions were pooled, pelleted, resuspended in buffer 2, and stored at −80 °C.

### Cryo-electron microscopy

UltrAuFoil^®^ grids (Quantifoil, R1.2/1.3) were glow-discharged for 20 s with a Solarus 950 (Gatan). In total, 3 μl of 70S complexes at 100 nM were placed on grids at 8 °C in 100% humidity and blotted for 3.5 s using a Vitrobot Mark IV (FEI).

Two independent datasets of 2,394 micrographs total (1863 and 531 micrographs, respectively) were collected on a Titan Krios (FEI) microscope operated at 300 kV with a C2 aperture diameter of 70 μm. Movie frames were recorded at an accumulated dose of 58 e^_^/A^°2^ at a magnification of 59,000X (corresponding to a pixel value of 1.191 Å) with a DE-64 direct electron detector in counting mode^64^ using Leginon^65^ for automatic data acquisition. Images were recorded with a total exposure time of 19.3 s, and intermediate frames were recorded every 0.2 s giving a total of 78 frames per image. Defocus values ranged from −1.3 to −3 μm.

### Image processing

Pre-processing — All processing steps were carried out using Appion^66^. All frames of each micrograph were aligned using MotionCor2^67^. Contrast transfer function (CTF) parameters were estimated on all motion-corrected micrographs using CTFFIND4^68^ and GCTF^67^ and the best estimate chosen using resolution evaluation in Appion^69^. 197 micrographs were excluded after manual inspection of corresponding power spectra displayed ice contamination, exposure to the shifted beam, or low-resolution Thon ring profiles. An initial set of ~2000 particles were picked using DoG (Difference of Gaussian) Picker^70^. A rotational average was generated from these picks, and this was used as a template for template-based picking using FindEM^71^. A total of 474,382 particles (373,845 and 100,537 particles from the first and the second dataset, respectively) were picked. Particles were extracted with a box size of 384 × 384 pixels in Appion.

Extracted particles from the two datasets were processed independently and combined after the last round of 3D reconstruction, and subjected to further 3D classifications and refinements in RELION-3^72^ (Supplementary Fig. 2). Processing steps were initially performed on a 4X-binned dataset.

Initial 3D refinement occurred against a 60 Å low-pass filtered empty *E. coli* 70S ribosome. 3D classification without alignment was used to discard free 50S subunits. The resulting 70S classes were combined and refined using an initial angular sampling of 7.5° and local angular sampling of 1.8° to improve angular assignment. Following refinement, another round of 3D classification without alignment was performed, with low resolution particles and particles containing E-site tRNA discarded. Two classes with an unrotated 70S were combined and subjected to refinement, followed by focused classification with a P-site mask. The P-site mask was generated from a 70S ribosome with P-site tRNA (PDB 4V4I)^73^, with a 5-voxel expansion and a 7-voxel soft edge. This resulted in a class of 70S particles with P-site tRNA. These particles were unbinned, refined, then underwent focused classification with an A site mask, created as stated, using a model of the 70S ribosome with A-site tRNA (PDB 4V5D)^23^. Classes with P-site tRNA but no A-site tRNA were refined and post-processed.

### Post-processing and beam-tilt correction

The resultant map was post-processed in RELION-3 using a solvent mask generated from the final reconstruction low-pass filtered to 40 Å with a 7-pixel extension and 10-pixel soft edge. Beam-tilt estimation and correction was performed (without per-particle refinement of CTF parameters) using CTF refinement followed by 3D reconstruction. These steps were repeated iteratively until the highest resolution was achieved, and the final map post-processed with a solvent mask. Resolution estimations were calculated from Fourier shell correlations (FSC) at 0.143 between the two independently refined half-maps. Maps were sharpened in PHENIX^74^. The graphs of directional 3D FSC and global resolution of the maps were plotted using 3DFSC Processing Server^75^. Local resolution was estimated using blocres in Bsoft^76^.

### Modeling

For initial model building, the coordinates of *E. coli* 70S ribosome (PDB 5MDV) were removed of P-site tRNA, E-site tRNA and mRNA and rigid-docked into cryo-EM maps using UCSF Chimera^77^. Ligand coordinates were initially generated in COOT using Lidia^78,79^. Ligand restraints were generated and edited in PHENIX eLBOW and REEL respectively^80^. P-site tRNA, mRNA, the N-terminus of bL27 and KKL-2098 coordinates were manually fit using COOT^79^. The P-site tRNA in PDB 5MDV was manually mutated to tRNA^Gly^, the last codon in the mRNA sequence. The assembled complex was then subjected to real space rigid body refinement followed by validation in PHENIX 1.17.1^80^.

To model KKL-2098 into the map, two possible conformations were considered as potential orientation. The final placement was selected for three reasons. The most important reason was the orientation of the photoreactive azide linkage which cross-linked previously to 23S rRNA 2452^11^. At the other end of the molecule, Zone 1 was the most tolerant of substitutions (Fig. 1) and its current placement indicated few interactions with the ribosome. Lastly, Zone 2 containing the oxadiazole was the least tolerant of changes and when Zone 1 and Zone 4 (containing the azide linkage) were positioned according to the first two rationales, KKL-2098 could make interactions with 23S rRNA A2602.

For the figures that show segmented maps (Supplementary Figs. 3D, 4, 5A,B, and 7A), the EM density map was low pass filtered to 5 Å for the areas which were at an overall lower resolution, including the P-tRNA and KKL-2098 and N-terminus of L-27. The resulting EM density was segmented using a 2.5–3.5 Å radius mask around the desired residues in Chimera^77^. The segmented densities were exported to ChimeraX for enhancing graphical features^81^. For panels that include, 16S, L20, and 23S rRNA the sharpened map was used since they were higher resolution features. Other figures were made using the PyMOL Molecular Graphics System, Version 2.1 (Schrödinger, LLC).

### Reporting summary

Further information on research design is available in the Nature Research Reporting Summary linked to this article.

## Supplementary information

Supplementary Information

Description of Additional Supplementary Files

Supplementary Movie 1

Reporting Summary

## Data Availability

The data that support this study are included in this published article and its supplementary information files, or are available from the authors on reasonable request. Structural data have been deposited with PDB accession code 6OM6 and EMDB with accession code EMD-20121. Source data is provided with this article and is also available at 10.26207/p6nj-8v13.

## Source data

Source Data
