# Supplementary material for: trans-Translation inhibitors bind to a novel site on the ribosome and clear Neisseria gonorrhoeae in vivo
Source: Nat Commun. 2021 Mar 19;12:1799. doi: 10.1038/s41467-021-22012-7 (PMC7979765; doi:10.1038/s41467-021-22012-7)
Supplement: Supplementary file 1 — Supplementary Information [file 41467_2021_22012_MOESM1_ESM.pdf]

trans-Translation inhibitors bind to a novel site on the ribosome and clear *Neisseria gonorrhoeae in vivo*

Zachary D. Aron[1,†], Atousa Mehrani[2,†], Eric D. Hoffer[3,†], Kristie L. Connolly[4], Pooja Srinivas[3,5], Matthew C. Torhan[1], John N. Alumasa[6], Mynthia Cabrera[6], Divya Hosangadi[6], Jay S. Barbor[1], Steven C. Cardinale[1], Steven M. Kwasny[1], Lucas R. Morin[1], Michelle M. Butler[1], Timothy J. Opperman[1], Terry L. Bowlin[1], Ann Jerse[4], Scott M. Stagg[2,7], Christine M. Dunham[3,*], Kenneth C. Keiler[6,*]

[1]Microbiotix, Inc. One Innovation Dr., Worcester, MA 01605, USA

[2]Department of Chemistry and Biochemistry, Florida State University

[3]Department of Biochemistry and Emory Antibiotic Resistance Center, Emory University School of Medicine

[4]Department of Microbiology and Immunology, Uniformed Services University, Bethesda, MD

[5]Molecular & Systems Pharmacology Graduate Program, Emory University

[6]Department of Biochemistry & Molecular Biology, Penn State University

[7]Institute of Molecular Biophysics, Florida State University

*Correspondence to: kkeiler@psu.edu, cmdunha@emory.edu

†These authors contributed equally

PDB accession code: 6OM6; EM accession code: EMD-20121

Corresponding Author: Kenneth Keiler, 401 Althouse Laboratory, Penn State University, University Park, PA 16802; (814) 863-0787; kkeiler@psu.edu
Christine Dunham, Emory University School of Medicine, 1510 Clifton Road NE, Room G223, Atlanta, GA 30322; (404)-712-1756; cmdunha@emory.edu

Keywords: ribosome, *trans*-translation, tmRNA, gonorrhea, antibiotic

Disclaimer: The contents of this article are solely the responsibility of the authors and do not necessarily represent the official views of the Department of Defense or the Uniformed Services University

SUPPLEMENTARY NOTES

Structure-Activity Relationships

Compound optimization studies first focused on evaluation of overall structure activity relationships (SAR), resulting in a large data set, a selection of which is shown in Supplementary Table 1. Analyses focused on the 4 conceptual zones of the molecule (Figure 1A). Although only partial details on SAR data are included here, a more detailed analysis will be the topic of a future publication.

Zone 1 was tolerant of aliphatic, aromatic and heteroaromatic groups (entries 1-3, Supplementary Table 1; heteroaromatics not shown), but complete replacement of the Zone 1 substituent with a hydrogen atom was not tolerated (entry 4, Supplementary Table 1). It should be noted that, although incorporating aliphatic groups in Zone 1 moderately increased potency, extensive oxidative metabolism at this site limited further work in this direction (not shown).

The oxadiazole ring (Zone 2) was critical to activity, with only oxadiazoles and oxazoles displaying measurable, if widely varied, activity, despite evaluation of >20 heterocycles (representative examples include entries 1, 5-6, Supplementary Table 1). This high level of specificity is consistent with hydrogen-bonding and/or dipole-directed interactions with this moiety in the binding site, as these properties vary widely among oxadiazoles [1].

Efforts to identify amide isosteres or alternatives in Zone 3 that avoid the observed amidolysis in KKL-35 proved challenging because variation of the amide was poorly tolerated. Of >10 isosteres examined, only analogs containing amides, ureas or *N*-alkyl amides were tolerated (entries 1, 7-9, and 12-16, Supplementary Table 1). This specificity is consistent with binding interactions involving the oxygen of the Zone 2 amide and a requirement for planarity in this region of the molecule. Although replacement of the amide with a urea prevented amidolysis, simple ureas such as MBX-4346 (entry 12, Supplementary Table 1) still demonstrated limited microsomal stability, consistent with aliphatic ring oxidation.

Variations in Zone 4 examined both amides and ureas. Among the amides, changes that disrupt coplanarity with the Zone 3 amide were poorly tolerated (*e.g.* aliphatics, entry 10, Supplementary Table 1; or C-2 substituents, not shown). Aromatic groups were well tolerated in Zone 4, with hydrophobic substituents distal to the core leading to improved potency (not shown). Among the ureas, small rings were moderately tolerated, with 6- and 7-membered rings providing improved potency relative to 5 membered rings (entries 12-14, Supplementary Table 1). Hydrophilic groups, such as the ether moiety in the morpholine ring of MBX-4697 (entry 15, Supplementary Table 1) had a deleterious effect on potency, although they dramatically increased

microsomal stability, consistent with limiting aliphatic group oxidation. Spirocyclic and bridged bicyclic rings had a significantly deleterious effect on potency, consistent with a need for a relatively flat cross-section in this region of the molecule (not shown). Fused bicyclic groups were generally well tolerated, provided they did not include branching on the carbon directly attached to the urea moiety; of these, fused aryl rings provided excellent enhancement in both potency and metabolic stability, resulting in the identification of MBX-4132 (entry 16, Supplementary Table 1).

Throughout SAR studies, *in vitro* ADME properties such as solubility, microsomal stability and cytotoxicity were monitored. Most compounds exhibited cytotoxicity profiles favorable for development and moderate-to-good solubility, likely due to the hydrophilic 1,3,4-oxadiazole moiety [1]. An initial concern was metabolic stability, with the Zone 3 amide providing a particular liability. Replacement of this group with a urea moiety provided a clear solution to the problem (entries 12-16, Supplementary Table 1). Further evaluation of MBX-4132 focused on multispecies microsomal stability, serum effects and permeability (Supplementary Table 2). These studies revealed excellent drug-like properties and predicted the observed oral bioavailability. It should be noted that MBX-4132 is highly serum-bound, a feature that has an impact on its potency in the presence of serum (Supplementary Table 2).

SUPPLEMENTARY TABLES AND FIGURES

Supplementary Table 1: Properties of acylaminooxadiazoles (subset of SAR analogs).

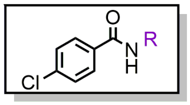

#	CMPD#	R	Luc IC_{50}[a]	ΔtolC *E. coli* MIC[b]	*Ng* MIC[b]	CC_{50}[c]	Sol[d]	MLMS[e]
1	KKL-35/ MBX-3535	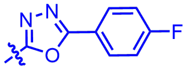	0.23	0.49	0.12	>100	6.25	<5
2	MBX-3910	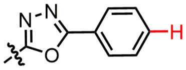	0.14	1.9	0.30	40	13	5
3	MBX-4370	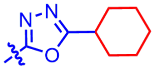	0.13	0.48	0.12	17	50	<5
4	MBX-4367	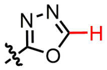	>25	22.3	11.2	>100	>200	<5
5	MBX-4083	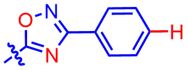	1.4	2.7	4.8	>100	6.3	<5
6	MBX-3943	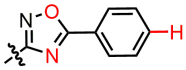	>25	>30	15.0	>100	13	<5

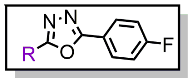

#	CMPD#	R	Luc IC_{50}[a]	ΔtolC *E. coli* MIC[b]	*Ng* MIC[b]	CC_{50}[c]	Sol[d]	MLMS[e]
7	MBX-3776	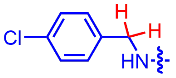	>25	>30	>30	>100	25	5
8	MBX-4076	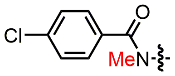	1.05	4.6	2.7	95	<3.1	
9	MBX-4063	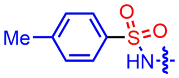	>25	>33	>33	>100	100	<5
10	MBX-3709	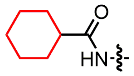	12.5	28.9	4.6	>100	25	<5
11	MBX-C4227	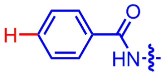	1.0	0.88	1.1	>100	50	<5
Urea Variants								
12	MBX-4346	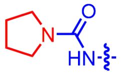	14.3	27.6	4.4	>100	>200	25
13	MBX-4699	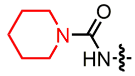	2.1	7.3	0.58	>100	50	12
14	MBX-4700	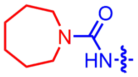	1.8	4.8	0.61	>100	25	19
15	MBX-4697	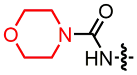	9.2	29.2	4.7	>100	100	>120
16	MBX-4132	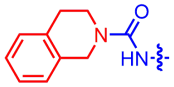	0.19	2.7	0.18	45	100	>120

[a] Half Maximal activity in ΔtolC *E. coli* luciferase assay (μM) [b] MIC vs *Neisseria gonorrhoeae* (49226) or *E. coli* KLE701 in μg/mL. [c] Against HeLa cells (μM). [d] Solubility in water (μM; nephlometry). [e] Murine liver microsome stability, $t_{1/2}$ in min at 37 °C in the presence of NADPH.

Supplementary Table 2: In Vitro ADME Properties

	Microsomal Stability ($t_{1/2}$; min)				serum		Serum Binding (% bound)				Caco-2 ($P_{app;}$ x 10^{-6} cms^{-1})	
	Murine	Rat	Dog	Hum.	Shift[a]	Stab[b]	Murine	Rat	Dog	Human	A→B	B→A
MBX-4132	>120	>120	>120	>120	16	>99.8	98.0	93.0	95.2	99.0	11.1	7.3

[a]Ratio of MIC +/- 10% fetal bovine serum added. [b]% remaining after 1 h incubation at 37 ˚C.

Supplementary Table 3: Anti-gonococcal spectrum

Strain	Resistance/Description	MIC (µg/ml)	
		KKL-35	MBX-4132
ATCC 49226	type strain	0.12	0.13
ATCC 700719	SPT	0.25	0.21
ATCC 700825	STR	0.06	0.04
BAA-1846	TET	0.06	0.04
HO41	PEN, TET, CFM, CRO, LVX	0.25	0.17
CDC-0165	CIP, PEN, TET	n.d.	0.68
CDC-0166	CIP, PEN, TET	n.d.	0.17
CDC-0167		n.d.	0.13
CDC-0169	CIP, PEN, TET	n.d.	0.17
CDC-0170	CIP, PEN, TET	n.d.	0.17
CDC-0171	CIP, PEN, TET	n.d.	0.17
CDC-0172	CIP, PEN, TET	n.d.	0.17
CDC-0173	CIP, PEN, TET	n.d.	0.17
CDC-0174	CIP, PEN, TET	n.d.	0.17
CDC-0175	CIP, PEN, TET	n.d.	0.08
CDC-0176		n.d.	0.34
CDC-0177	CIP, PEN, TET	n.d.	0.17
CDC-0178	TET	n.d.	0.34
CDC-0179	CIP, PEN, TET	n.d.	0.08
CDC-0180		n.d.	0.17
CDC-0181	CIP, PEN, TET	n.d.	0.17
CDC-0182	TET	n.d.	0.17
CDC-0183	CIP, PEN, TET	n.d.	0.34
CDC-0184	CIP, PEN, TET	n.d.	0.17
CDC-0185	CIP, PEN, TET	n.d.	0.27
CDC-0186	CIP, PEN, TET	n.d.	0.17
CDC-0187	CIP, PEN, TET	n.d.	0.34
CDC-0188	CIP, PEN	n.d.	0.17
CDC-0189	CIP, PEN, TET	n.d.	0.13
CDC-0190	CIP, PEN, TET	n.d.	0.17
WHO F		n.d.	0.06
WHO G	PEN, TET	n.d.	0.13
WHO K	CIP, PEN, TET	n.d.	0.26
WHO L	CIP,PEN, TET	n.d.	0.26
WHO M	CIP,PEN,TET	n.d.	0.13
WHO N	CIP, PEN, TET	n.d.	0.26

WHO O	PEN, TET	n.d.	0.13
WHO P	PEN, TET	n.d.	0.33
WHO U	PEN, TET	n.d.	0.13
WHO V	AZM, CIP, PEN, TET	n.d.	0.26
WHO W	CIP, PEN, TET	n.d.	0.13
WHO X	CIP, PEN, TET	n.d.	0.21
WHO Y	CIP, PEN, TET	n.d.	0.21
WHO Z	CIP, PEN, TET	n.d.	0.26
MMX ATCC 49226		0.25	0.27
MMX 6744		0.13	0.27
MMX 6746	CIP	0.13	0.14
MMX 6752	TET	0.25	0.27
MMX 6753		0.06	0.14
MMX 6757		0.06	0.07
MMX 6758		0.13	0.27
MMX 6762		0.13	0.27
MMX 6767		0.06	0.14
MMX 6771	TET	0.06	0.14
MMX 6793	CIP	0.06	0.14
MMX 6797	CIP, TET	0.06	0.14
MMX 6803	CIP	0.13	0.27
MMX 6812	CIP	0.13	0.27
MMX 6818	CIP	0.13	0.27
MMX 6819	CIP	0.13	0.14
MMX 6879		0.06	0.14
MMX 6921	CIP	0.25	0.53
MMX 6922	CIP	0.25	0.53
MMX 6983	CIP	0.25	0.53
MMX 6989		0.13	0.14
MMX 6990	CIP	0.06	0.14
MMX 6992		0.13	0.14
MMX 6996		0.13	0.27
MMX 6998	CIP	0.25	0.53
MMX 7002	CIP	0.13	0.27
MMX 7005		0.03	0.03
MIC_{90} (n)		0.25 (32)	0.53 (71)
MIC Range (n)		0.03-0.25 (32)	0.03-0.68 (71)

SPT, spectinomycin; STR, streptomycin; TET, tetracycline; PEN, penicillin G; CFM, cefixime; CRO, ceftriazone; CIP, ciprofloxacin; LVX, levofloxacin; AZM, azithromycin; MMX, Micromyx, LLC strain (these MIC assays were performed, using the broth dilution method, by Micromyx, LLC.)

Supplementary Table 4. Antibacterial spectrum

Category	Organism	Strain	Resistance/Description	MIC (μg/ml) KKL-35	MIC (μg/ml) MBX-4132
Gram-negative	*Escherichia coli*	KLE700		≥32	≥34
	Escherichia coli	KLE701	KLE700 *ΔtolC::tet*	0.5	2.8
	Klebsiella pneumoniae	ATCC 13883		≥32	≥34
	Acinetobacter baumannii	ATCC 19606		>32	≥34
	Pseudomonas aeruginosa	ATCC 27853		>32	≥34
	Moraxella catarrhalis	8716		n.d.	0.04
	Legionella pneumophila	ATCC 33153		n.d.	8.5
	Haemophilus influenzae	ATCC 35056		n.d.	17.5
Gram-positive	*Staphylococcus aureus*	BAA-1717	MRSA	1	1
	Staphylococcus aureus	MRSA-1234547263	MRSA	1	3.2
	Staphylococcus aureus	MRSA-1094	MRSA	1	3.2
	Staphylococcus aureus	ATCC 35556		1	4.2
	Staphylococcus aureus	NRS-77		1	3.2
	Staphylococcus aureus	MSSA		1	3.2
	Staphylococcus aureus	N315, NRS-70	MRSA	1	6.3
	Staphylococcus aureus	ATCC 25923		1	6.3
	Staphylococcus aureus	THC1516	MRSA	0.9	1
	Streptococcus pneumoniae	ATCC 49619		n.d.	34
	Mycoplasma pneumoniae	ATCC 15531		n.d.	≥34

Supplementary Table 5. Eurofins Discovery Services *In Vitro* Safety Panel, Cyp inhibition assays and Cardiac Ion Channel interaction results for MBX-4132.

Catalog ref[A]	Receptor Profiling	Species	Conc	% inhibition
104010	Cholinesterase, Acetyl, ACES	hum	10 μM	6
116030	Cycloogenase COX-1	hum	10 μM	8
118030	Cycloogenase COX-2	hum	10 μM	10
140010	Monoamine Oxidase MAO-A	hum	10 μM	-108
152300	Phosphodiesterase PDE3A	hum	10 μM	-6
154420	Phosphodiesterase PDE4D2	hum	10 μM	3

176020	Protein Tyrosine Kinase, LCK	hum	10 μM	29
200610	Adenosine A_{2A}	hum	10 μM	42
203110	Adrenergic α_{1A}	hum	10 μM	6
203630	Adrenergic α_{2A}	hum	10 μM	35
204010	Adrenergic β_1	hum	10 μM	-8
204110	Adrenergic β_2	hum	10 μM	35
206000	Androgen (testosterone)	hum	10 μM	-2
214600	Calcium Channel L-Type, Dihydropyridine	rat	10 μM	11
217050	Cannabinoid CB_1	hum	10 μM	-4
217100	Cannabinoid CB_2	hum	10 μM	-4
218030	Cholecystokinin CKK_1 (CCK_A)	hum	10 μM	66
219500	Dopamine D_1	hum	10 μM	26
219700	Dopamine D_{2s}	hum	10 μM	21
224010	Endothelin ET_A	hum	10 μM	-15
226600	$GABA_A$, Flunitrazepam, Central	rat	10 μM	3
232030	Glucocorticoid	hum	10 μM	-2
232810	Glutamate, NMDA, Agonism	rat	10 μM	8
239610	Histamine H_1	hum	10 μM	11
239710	Histamine H_2	hum	10 μM	-7
252610	Muscarinic M_1	hum	10 μM	-7
252710	Muscarinic M_2	hum	10 μM	7
252810	Muscarinic M_3	hum	10 μM	5
299031	Nicotinic Acetylcholine α4β2, Cytisine	hum	10 μM	-5
260130	Opiate $Delta_1$ (OP1, DOP)	hum	10 μM	5
260210	Opiate Kappa (OP2, KOP)	hum	10 μM	13
260410	Opiate Mu (OP3, MOP)	hum	10 μM	17
265510	Potassium Channel [K_A]	rat	10 μM	-2
265910	Potassium Channel hERG, [^{3}H Dofetilide	hum	10 μM	27
271110	Serotonin (5-hydroxytryptamine) $5\text{-}HT_{1A}$	hum	10 μM	30
271230	Serotonin (5-hydroxytryptamine) $5\text{-}HT_{1B}$	hum	10 μM	21
271650	Serotonin (5-hydroxytryptamine) $5\text{-}HT_{2A}$	hum	10 μM	18

271700	Serotonin (5-hydroxytryptamine) 5-HT_{2B}	hum	10 µM	44
271910	Serotonin (5-hydroxytryptamine) 5-HT_{3}	hum	10 µM	4
279510	Sodium Channel, Site 2	rat	10 µM	21
220320	Transporter, Dopamine (DAT)	hum	10 µM	3
204410	Transporter, Norepinephrine (NET)	hum	10 µM	6
274030	Transporter, Serotonin (5-Hyroxytryptamine) (SERT)	hum	10 µM	-10
287530	Vasopressin V_{1A}	hum	10 µM	-4

Catalog ref[A]	Cyp Inhibition Assays	Species	pIC_{50}
4876	CYP1A inhibition (Phenacetin substrate)	hum	NC[B]
4877	CYP2B6 inhibition (Bupropion substrate)	hum	>100 µM
4879	CYP2C8 inhibition (Amodiaquine substrate)	hum	>100 µM
4878	CYP2C9 inhibition (Diclofenac substrate)	hum	NC[B]
4874	CYP2C19 inhibition (Omeprazole substrate)	hum	>100 µM
4875	CYP2D6 inhibition (Dextromethorphan substrate)	hum	>100 µM
4873	CYP3A inhibition (Midazolam substrate)	hum	NC[B]
4872	CYP3A inhibition (Testosterone substrate)	hum	>100 µM

Catalog ref[A]	Cardiac Ion Channel Panel	Mode	Est. IC_{50} (µM)
CYL8004QP2DR	Nav1.5	Antagonist	>30
CYL8038QP2DR	hERG	Antagonist	>30
CYL8007QP2DR	KCNQ1/mink	Antagonist	>30
CYL8069QP2DR	Kv4.3/ChIP2	Antagonist	>30 (Peak&End)
CYL8032QP2DR	Kir2.1	Antagonist	>30 (Peak&End)
CYL8051QP2DR	Cav1.2	Antagonist	>30
CYL7004QP1DR	Nav1.5 late current	Agonist	>30
CYL7004QP2DR	Nav1.5 late current	Antagonist	>30

[A]Assays were performed using established protocols by Eurofins Discovery Services; catalog references provided. [B]No activity observed at any tested concentration.

Supplementary Table 6. Evaluation of MBX-4132 in the *Salmonella*/Microsome Plate Incorporation Assay (AMES Screen) performed by SRI Biosciences, a division of SRI International.

Strain	Test Compound/Condition	Dose (μg/plate)	Mean Revertants/plate
TA98	DMSO	N/A	23.7 ± 6.7
TA100	DMSO	N/A	127.3 ± 4.5
TA98	DMSO+S9	N/A	28.7 ± 5.9
TA100	DMSO+S9	N/A	129.0 ± 4.4
TA98	2-Nitrofluorene	5	1331.3 ± 171.6
TA100	Sodium Azide	5	2029.0 ± 49.6
TA98	2-Aminoanthracene + S9	2	1206.7 ± 169.1
TA100	2-Aminoanthracene + S9	2	1438.7 ± 55.8
TA98	MBX-4132	1	21.5 ± 2.1
TA98	MBX-4132	5	17.0 ± 1.4
TA98	MBX-4132	10	15.0 ± 1.4
TA98	MBX-4132	50	20.0 ± 2.8
TA98	MBX-4132	100	20.0 ± 2.8
TA98	MBX-4132	500	17.5 ± 2.1
TA100	MBX-4132	1	112.5 ± 6.4
TA100	MBX-4132	5	99.0 ± 1.4
TA100	MBX-4132	10	97.0 ± 4.2
TA100	MBX-4132	50	73.0 ± 8.5
TA100	MBX-4132	100	54.0 ± 1.4
TA100	MBX-4132	500	35.5 ± 3.5
TA98	MBX-4132 + S9	1	23.5 ± 2.1
TA98	MBX-4132 + S9	5	22.0 ± 0.0
TA98	MBX-4132 + S9	10	21.0 ± 2.8
TA98	MBX-4132 + S9	50	18.5 ± 0.7
TA98	MBX-4132 + S9	100	17.5 ± 3.5
TA98	MBX-4132 + S9	500	20.0 ± 2.8
TA100	MBX-4132 + S9	1	107.5 ± 4.9
TA100	MBX-4132 + S9	5	125.5 ± 7.8
TA100	MBX-4132 + S9	10	102.0 ± 15.6
TA100	MBX-4132 + S9	50	71.0 ± 4.2
TA100	MBX-4132 + S9	100	43.0 ± 4.2
TA100	MBX-4132 + S9	500	19.5 ± 0.7

Supplementary Table 7. Results from mitchondrial toxicity studies examining the effect of MBX-4132 on reactive oxygen species (ROS), mitochondrial membrane potential (MMP) and differential cytotoxicity against HepG2 cells grown on glucose or galactose.

Compound	Conc. (µM)	Attached live cells (%)	ROS (fold induction)	MMP inhibition (fold induction)
MBX-4132	3.18E-03	105.6 ± 7.5	3.1 ± 4.1	1.0 ± 0.1
MBX-4132	1.01E-02	109.5 ± 8.4	1.4 ± 0.4	1.0 ± 0.1
MBX-4132	3.18E-02	97.5 ± 12.2	0.9 ± 0.5	0.9 ± 0.2
MBX-4132	1.00E-01	96.7 ± 6.9	1.4 ± 1.1	1.2 ± 0.3
MBX-4132	3.17E-01	114.0 ± 14.4	1.2 ± 0.5	0.9 ± 0.1
MBX-4132	1.00E+00	106.3 ± 3.3	1.2 ± 0.4	0.9 ± 0.1
MBX-4132	3.17E+00	99.6 ± 6.1	1.1 ± 0.3	1.0 ± 0.2
MBX-4132	1.00E+01	97.7 ± 9.4	1.9 ± 1.0	1.1 ± 0.1
MBX-4132	3.16E+01	89.5 ± 3.6	1.3 ± 0.6	1.1 ± 0.1
MBX-4132	1.00E+02	69.6 ± 12.3	9.2 ± 3.6	0.8 ± 0.1
Compound	Conc. (µM)	Cell type	Medium	% viability
MBX-4132	3.00E-06	HepG2	Glucose	109.6 ± 0.2
MBX-4132	1.00E-05	HepG2	Glucose	107.8 ± 2.1
MBX-4132	3.00E-05	HepG2	Glucose	106.5 ± 3.3
MBX-4132	1.00E-04	HepG2	Glucose	85.6 ± 1.8
MBX-4132	3.00E-06	HepG2	Galactose	94.1 ± 9.3
MBX-4132	1.00E-05	HepG2	Galactose	95.4 ± 0.7
MBX-4132	3.00E-05	HepG2	Galactose	96.8 ± 0.4
MBX-4132	1.00E-04	HepG2	Galactose	64.0 ± 10.3

Supplementary Table 8. Bacterial strains, plasmids, and synthetic sequences.

strain name	description	source
E. coli BL21 (DE3) pET28-H10arfArnc	Strain for expressing non-stop ribosomes	this work
IW312	*E. coli* LG90 Δ*rpmA*	[2]
IW312 pL27	contains plasmid for inducible expression of wild-type L27	[2]
IW312 pL27 –3	contains plasmid for inducible expression of L27 –3	[2]
IW312 pL27 –6	contains plasmid for inducible expression of L27 –6	[2]
IW312 Δ*tolC* pL27	IW312 pL27 with *tolC* deleted	this work
IW312 Δ*tolC* pL27 –3	IW312 pL27 –3 with *tolC* deleted	this work
IW312 Δ*tolC* pL27 –6	IW312 pL27 –6 with *tolC* deleted	this work
N. gonorrhoeae H041	Clinical multiple-antibiotic resistant isolate	[3]
N. gonorrhoeae	ATCC 49226	ATCC
N. gonorrhoeae	ATCC 700719	ATCC
N. gonorrhoeae	ATCC 700825	ATCC
N. gonorrhoeae	BAA-1846	ATCC
N. gonorrhoeae	CDC-0165	CDC-ARBank
N. gonorrhoeae	CDC-0166	CDC-ARBank
N. gonorrhoeae	CDC-0167	CDC-ARBank
N. gonorrhoeae	CDC-0169	CDC-ARBank
N. gonorrhoeae	CDC-0170	CDC-ARBank
N. gonorrhoeae	CDC-0171	CDC-ARBank
N. gonorrhoeae	CDC-0172	CDC-ARBank
N. gonorrhoeae	CDC-0173	CDC-ARBank
N. gonorrhoeae	CDC-0174	CDC-ARBank
N. gonorrhoeae	CDC-0175	CDC-ARBank
N. gonorrhoeae	CDC-0176	CDC-ARBank
N. gonorrhoeae	CDC-0177	CDC-ARBank
N. gonorrhoeae	CDC-0178	CDC-ARBank
N. gonorrhoeae	CDC-0179	CDC-ARBank
N. gonorrhoeae	CDC-0180	CDC-ARBank
N. gonorrhoeae	CDC-0181	CDC-ARBank
N. gonorrhoeae	CDC-0182	CDC-ARBank
N. gonorrhoeae	CDC-0183	CDC-ARBank
N. gonorrhoeae	CDC-0184	CDC-ARBank
N. gonorrhoeae	CDC-0185	CDC-ARBank
N. gonorrhoeae	CDC-0186	CDC-ARBank
N. gonorrhoeae	CDC-0187	CDC-ARBank
N. gonorrhoeae	CDC-0188	CDC-ARBank
N. gonorrhoeae	CDC-0189	CDC-ARBank
N. gonorrhoeae	CDC-0190	CDC-ARBank
N. gonorrhoeae	WHO F	CDC-ARBank
N. gonorrhoeae	WHO G	CDC-ARBank

N. gonorrhoeae	WHO K	CDC-ARBank
N. gonorrhoeae	WHO L	CDC-ARBank
N. gonorrhoeae	WHO M	CDC-ARBank
N. gonorrhoeae	WHO N	CDC-ARBank
N. gonorrhoeae	WHO O	CDC-ARBank
N. gonorrhoeae	WHO P	CDC-ARBank
N. gonorrhoeae	WHO U	CDC-ARBank
N. gonorrhoeae	WHO V	CDC-ARBank
N. gonorrhoeae	WHO W	CDC-ARBank
N. gonorrhoeae	WHO X	CDC-ARBank
N. gonorrhoeae	WHO Y	CDC-ARBank
N. gonorrhoeae	WHO Z	CDC-ARBank
N. gonorrhoeae	MMX 6744	Micromyx, LLC
N. gonorrhoeae	MMX 6746	Micromyx, LLC
N. gonorrhoeae	MMX 6752	Micromyx, LLC
N. gonorrhoeae	MMX 6753	Micromyx, LLC
N. gonorrhoeae	MMX 6757	Micromyx, LLC
N. gonorrhoeae	MMX 6758	Micromyx, LLC
N. gonorrhoeae	MMX 6762	Micromyx, LLC
N. gonorrhoeae	MMX 6767	Micromyx, LLC
N. gonorrhoeae	MMX 6771	Micromyx, LLC
N. gonorrhoeae	MMX 6793	Micromyx, LLC
N. gonorrhoeae	MMX 6797	Micromyx, LLC
N. gonorrhoeae	MMX 6803	Micromyx, LLC
N. gonorrhoeae	MMX 6812	Micromyx, LLC
N. gonorrhoeae	MMX 6818	Micromyx, LLC
N. gonorrhoeae	MMX 6819	Micromyx, LLC
N. gonorrhoeae	MMX 6879	Micromyx, LLC
N. gonorrhoeae	MMX 6921	Micromyx, LLC
N. gonorrhoeae	MMX 6922	Micromyx, LLC
N. gonorrhoeae	MMX 6983	Micromyx, LLC
N. gonorrhoeae	MMX 6989	Micromyx, LLC
N. gonorrhoeae	MMX 6990	Micromyx, LLC
N. gonorrhoeae	MMX 6992	Micromyx, LLC
N. gonorrhoeae	MMX 6996	Micromyx, LLC
N. gonorrhoeae	MMX 6998	Micromyx, LLC
N. gonorrhoeae	MMX 7002	Micromyx, LLC
N. gonorrhoeae	MMX 7005	Micromyx, LLC
Escherichia coli	KLE700	[4]
Escherichia coli	KLE701 *tolC::tet*	[4]
Klebsiella pneumoniae	ATCC 13883	ATCC
Acinetobacter baumannii	ATCC 19606	ATCC
Pseudomonas aeruginosa	ATCC 27853	ATCC
Moraxella catarrhalis	ATCC 8716	ATCC
Legionella pneumophila	ATCC 33153	ATCC

Haemophilus influenzae	ATCC 35056		ATCC
Staphylococcus aureus	BAA-1717		ATCC
Staphylococcus aureus	MRSA-1234547263		[5]
Staphylococcus aureus	MRSA-1094		[6]
Staphylococcus aureus	ATCC 35556		ATCC
Staphylococcus aureus	NRS-77		BEI Resources
Staphylococcus aureus	MSSA		[6]
Staphylococcus aureus	N315, NRS-70		BEI Resources
Staphylococcus aureus	ATCC 25923		ATCC
Staphylococcus aureus	THC1516		[7]
Streptococcus pneumoniae	ATCC 49619		ATCC
Mycoplasma pneumoniae	ATCC 15531		ATCC
plasmid name	description		source
pET28-H10arfArnc	plasmid to produce non-stop ribosomes *in vivo*		this work
pNL3.1	nano-luciferase encoding plasmid		Promega
pMC1	Nano-luciferase gene cloned into the NcoI and BamH1 sites of pET28		this work
DNA name	description	sequence	source
T7 universal	primer	TAATACGACTCACTATAGGG	ThermoFisher
nanoluc-ns	primer	CCCCCCGGTTACCCGGAAGA<br>GCAGGGAGCCGTC	this work
nanoluc-stop	primer	TTACAGAATCTCCTCGAACAG<br>CCG	this work
tmRNA-nl template	synthetic DNA cassette	GGGGCTGATTCTGGATTCGA<br>CGGGATTTGCGAAACCCAAG<br>GTGCATGCCGAGGGGCGGTT<br>GGCCTCGTAAAAAGCCGCAA<br>AAAATAGTCGCAGTCTCCGG<br>ATGGCGCCTTTTTAAAAAAAT<br>TTCTTAATAACAATTTTTTTAG<br>CCCTCTCTCCCTAGCCTCCG<br>CTCTTAGGACGGGGATCAAG<br>AGAGGTCAAACCCAAAAGAG<br>ATCGCGTGGAAGCCCTGCCT<br>GGGGTTGAAGCGTTAAAACTT<br>AATCAGGCTAGTTTGTTAGTG<br>GCGTGTCCGTCCGCAGCTGG<br>CAAGCGAATGTAAAGACTGA<br>CTAAGCATGTAGTACCGAGG<br>ATGTAGGAATTTCGGACGCG<br>GGTTCAACTCCCGCCAGCTC<br>CACCA	this work

A.

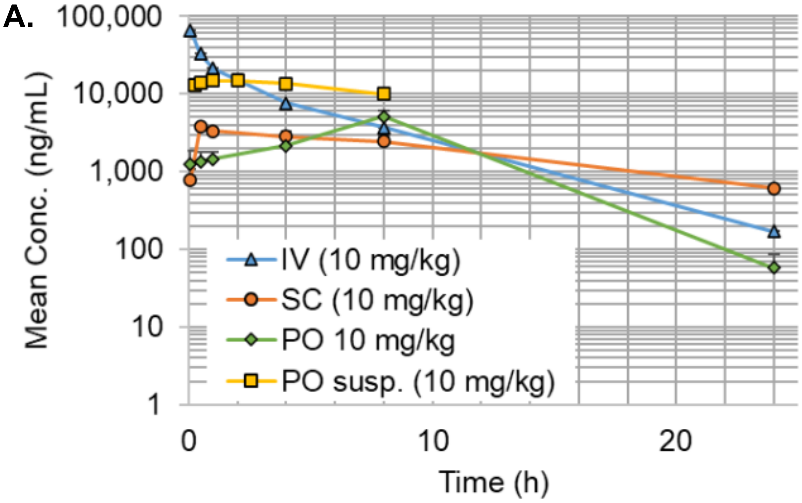

B.

	$T_{1/2}$ (hr)	$C_{0/max}$ (ng/mL)	AUC_{last} (hr*ng/mL)	V_{ss} (mL/kg)	Cl (mL/hr/kg)	%F (Last)
10 mg/kg IV	3.55	76,507	137,462	266	72.6	-
10 mg/kg SC	8.76	3,793	46,916	-	-	34
25 mg/kg SC	-	5,100	76,085	-	-	-
10 mg/kg PO	-	5,110	62,617	-	-	46
10 mg/kg susp. PO	-	14,812	181,695	-	-	>95*

Supplementary Figure 1. Pharmacokinetic properties of MXB-4132 (A) Graphical presentation of mean murine plasma concentration over time for MBX-4132. Error bars indicate standard deviation and are only shown in the positive direction for clarity. “susp.” refers to the suspension formulation studies performed at Neosome, all other data is from liquid formulation performed at Charles River Labs. (B) Calculated parameters for each dosing regimen of MBX-4132. %F for the 10 mg/kg PO suspension formulation is an estimate based on the IV data for different mouse species and sex. Source data are provided as a Source Data file.

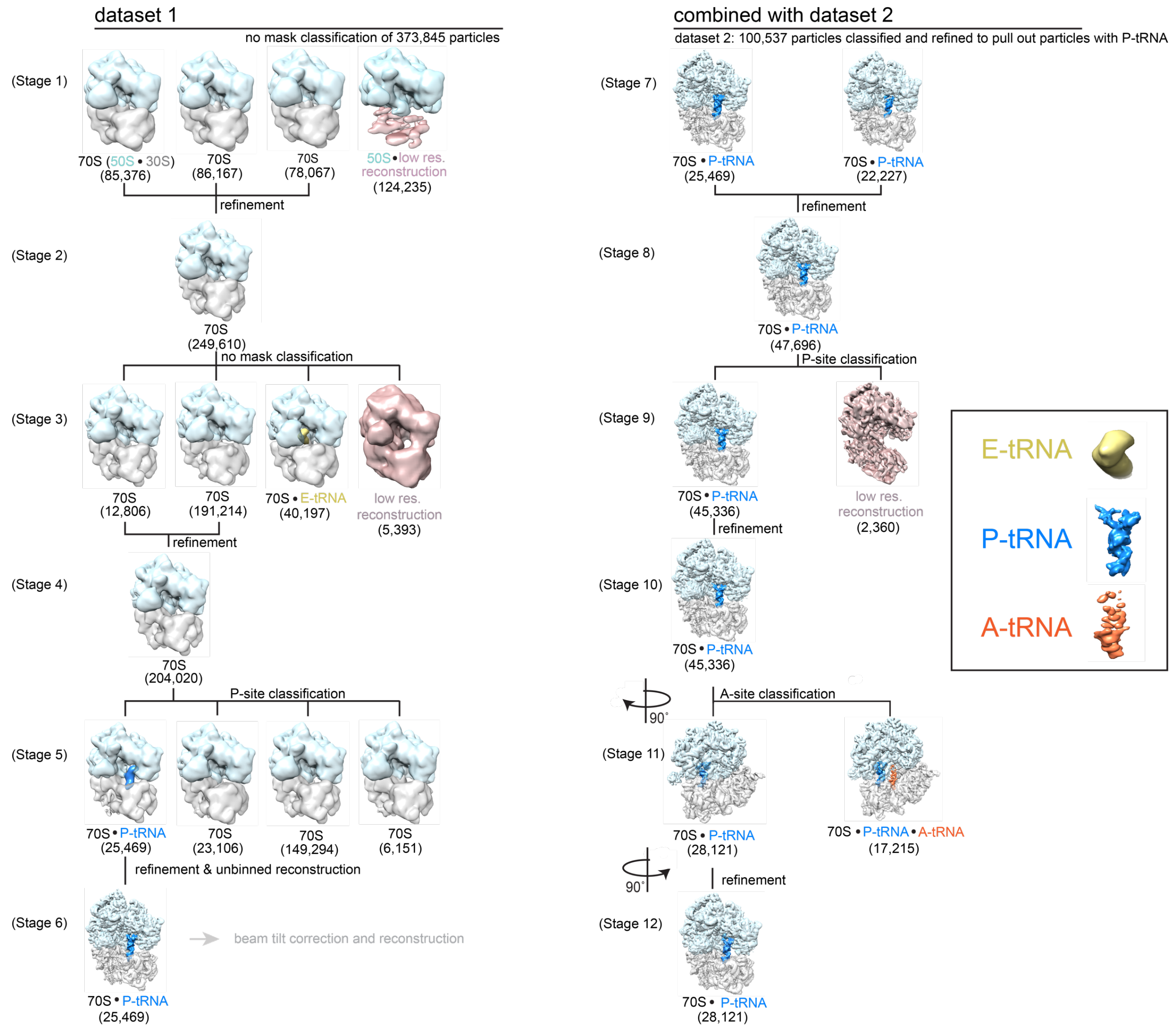

Supplementary Figure 2. Classification of cryo-EM datasets of *E. coli* 70S complex containing P-site tRNA and KKL-2098. To extract 70S particles with P-site tRNA and the KKL-2098 molecule several classification and refinement steps were performed. Stage 1) All particles were refined and reconstructed, then classification without alignment was performed to split the particles into 4 classes. Stage 2) 50S particles from stage 1 (shown in blue) were removed and the remaining particles refined. Stage 3) The aligned particles from stage 2 were classified into 4 classes. Stage 4) Particles contributing to the low-resolution reconstruction (pink) and 70S structures containing E-site tRNA (yellow) were removed and the remaining particles refined. Stage 5) Particles were classified without alignment into 4 classes using a P-site mask to identify particles with P-tRNA (blue). Stage 6) Particles containing P-tRNA were combined, unbinned and refined. Stage 7) Particles were beam-tilt corrected. Stage 8) Particles from both datasets were combined and refined together. Stage 9) The combined particles were classified using a P-site mask. Stage 10) Particles contributing to the lower-resolution reconstruction were eliminated and 70S/P-tRNA particles were refined. Stage 11) The reconstruction from stage 10 contained some A-site tRNA density, so a focused classification without alignment using an A-site mask was performed. Ribosomes are shown at 90° rotated view in respect to stage 10. Stage 12) 70S particles containing A-site tRNA (orange) were eliminated and 70S/P-tRNA particles were refined to an

overall resolution of 3.2 Å. The numbers of particles that make up each reconstruction are depicted for each complex.

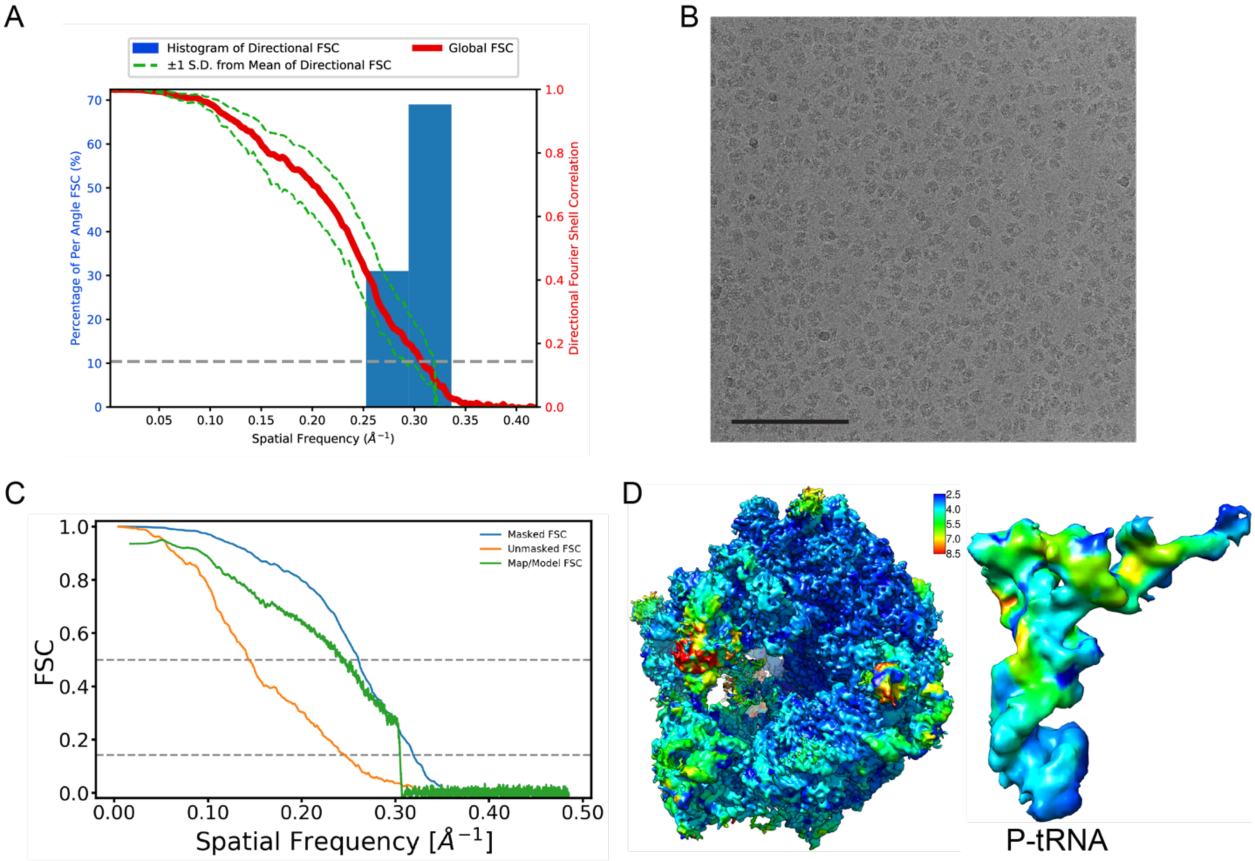

Supplementary Figure 3. Resolution characterization and quality of cryo-EM maps. (A) 3D FSC plot showing degree of directional anisotropy for the 70S•P-tRNA•KKL map. (B) A representative micrograph from the cryo-EM dataset. The scalebar represents 200 nm. (C) FSC plots for the 70S•P-tRNA•KKL map for the independent half maps (orange) masked (cyan), and map/model FSC (green). Dotted lines are shown at $FSC_{0.5}$ and $FSC_{0.143}$. (D) Local resolution estimate for the 70S-P-tRNA-KKL-2098 map estimated from blocres (left). The right panel shows the local resolution of the P-tRNA. The map is colored from highest resolution (blue) to lowest resolution (red).

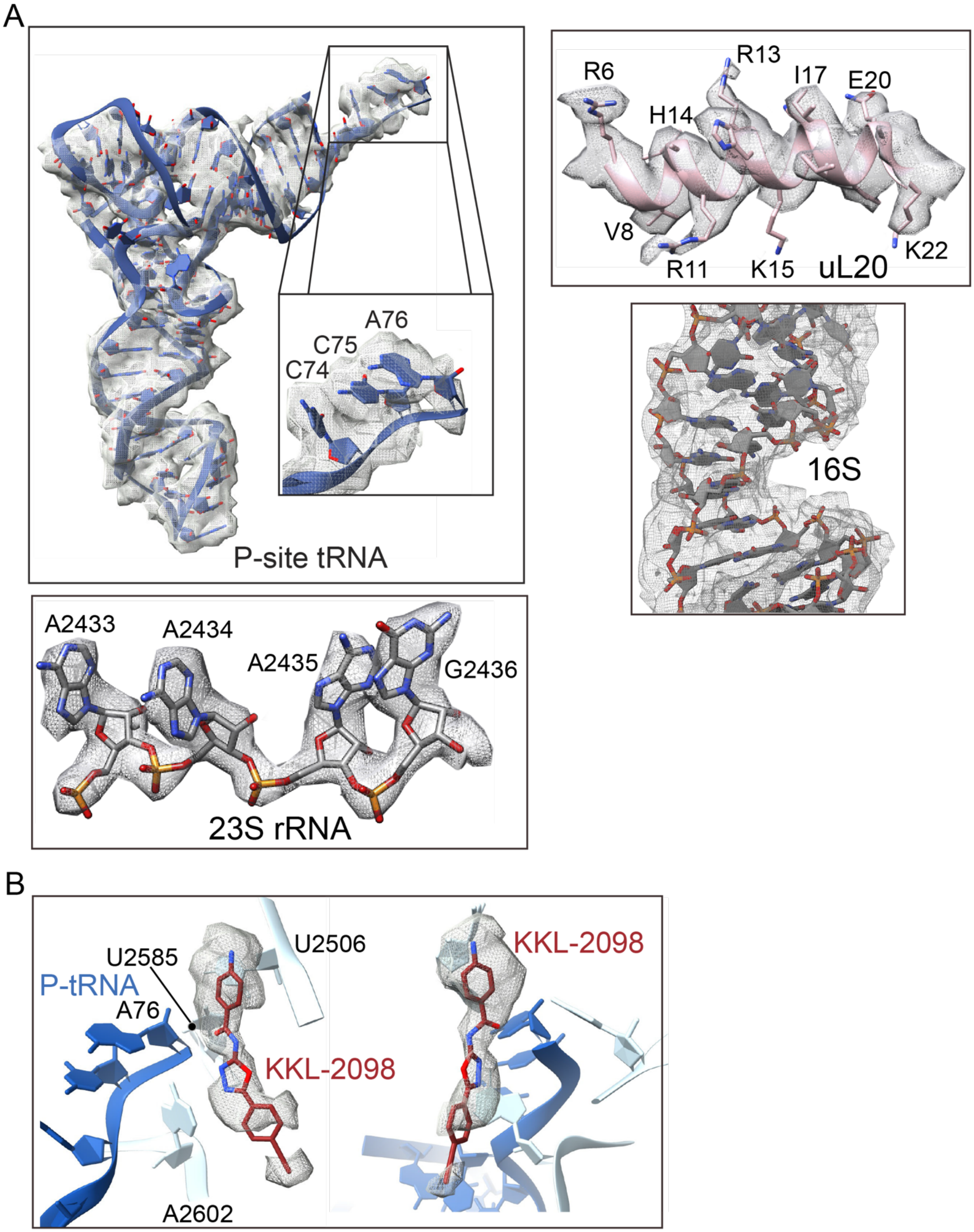

Supplementary Figure 4. Data quality of representative areas of the 70S-P-tRNA-KKL-2098 map. (A) Representative map quality of the P-site tRNA with the inset showing the CCA end. Map quality for ribosomal protein L20, 16S rRNA and 23S rRNA. (B) Map quality of KKL-2098.

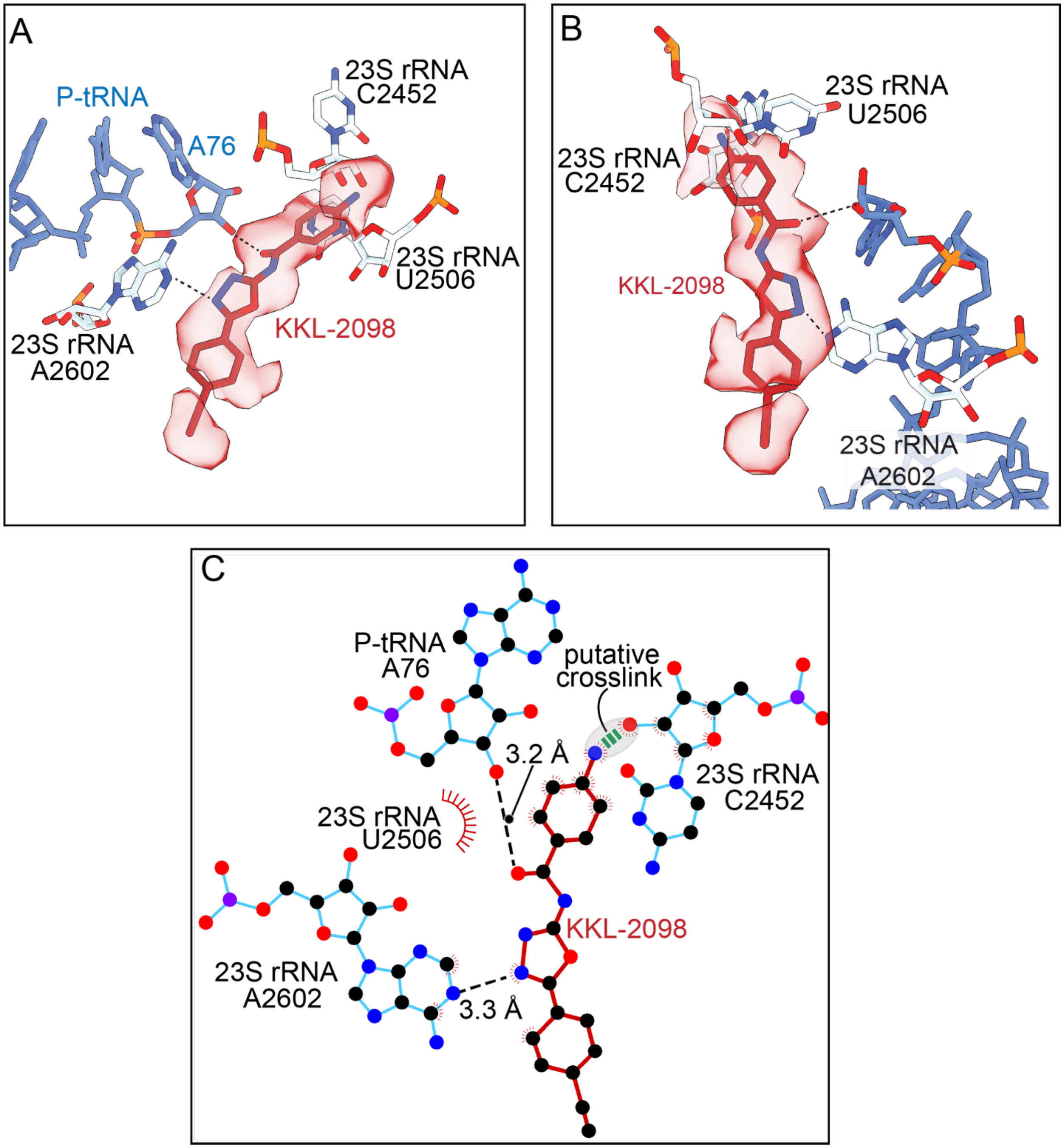

Supplementary Figure 5. Interaction network of KKL-2098 with the ribosome. (A) Interactions with of KKL-2098 with P-site tRNA A76 and 23 rRNA nucleotides C2452, U2506, and A2602 with a ~90º rotation in panel B. (C) A 2-dimensional representation of these interactions using LigPlot+.

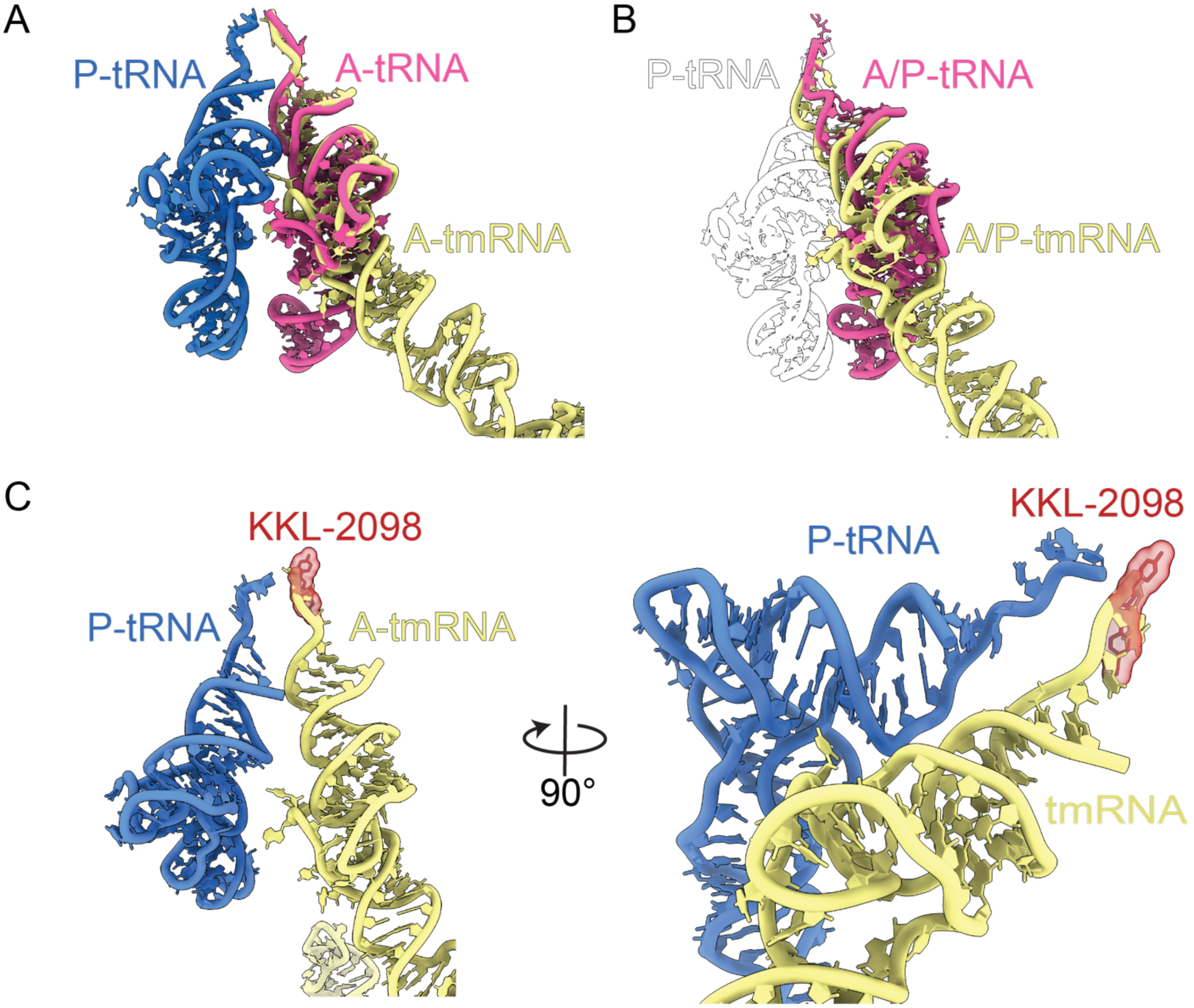

Supplementary Figure 6. Comparison of the position of KKL-2098 to A-site tRNA and A-site tmRNA. (A) Comparison of an A-site tRNA (PDB code 4V5D, https://www.rcsb.org/structure/4V5D) to an A-site tmRNA molecule (PDB code 6Q95, https://www.rcsb.org/structure/6Q95) reveals similarities in how both tRNAs interact with the PTC on the 50S. (B) Comparison of an A/P tRNA (PDB code 6WDF, https://www.rcsb.org/structure/6WDF) with an A/P tmRNA (PDB code 6Q97, https://www.rcsb.org/structure/6Q97) reveals slight differences in the interactions with the 50S but the major difference is that A/P tmRNA does not cause intersubunit rotation of the 50S relative to the 30S that normally accompanies hybrid state formation. (C) Overlay of A-site tmRNA (PDB code 6Q95, https://www.rcsb.org/structure/6Q95) with the structure in this study containing KKL-2098 reveals a steric clash between the two molecules.

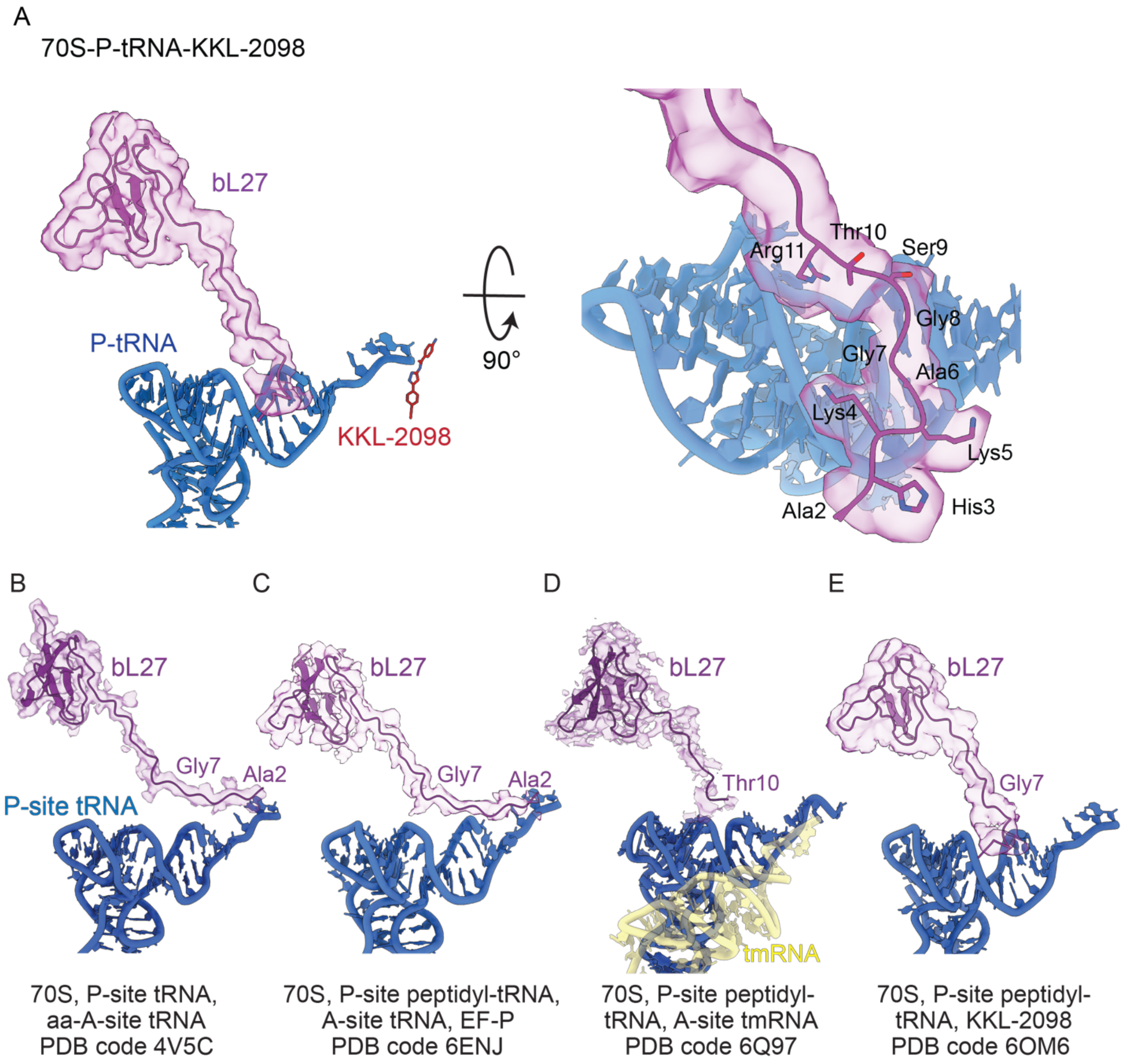

Supplementary Figure 7. bL27 map quality and its orientation in other structures. (A) In the 70S-P-site tRNA-KKL-2098 structure, the N terminus of bL27 (purple) moves ~180º away from the PTC. The previously observed position of bL27 is shown in green (PDB code 6ENU, https://www.rcsb.org/structure/6ENU). Position and electron potential maps of bL27 in PDB codes 4V5C https://www.rcsb.org/structure/4V5C (B), 6ENJ https://www.rcsb.org/structure/6Q97 (C), 6Q97 https://www.rcsb.org/structure/6Q97 (D) and 6OM6 https://www.rcsb.org/structure/unreleased/6OM6 (E).

SUPPLEMENTARY REFERENCES

1. Boström, J., Hogner, A., Llinàs, A., Wellner, E. & Plowright, A. T. Oxadiazoles in Medicinal Chemistry. *J. Med. Chem.* **55**, 1817–1830 (2012).
2. Maguire, B. A., Beniaminov, A. D., Ramu, H., Mankin, A. S. & Zimmermann, R. A. A Protein Component at the Heart of an RNA Machine: The Importance of Protein L27 for the Function of the Bacterial Ribosome. *Mol. Cell* **20**, 427–435 (2005).
3. Ohnishi, M. *et al.* Ceftriaxone-Resistant Neisseria gonorrhoeae, Japan. *Emerg. Infect. Dis.* **17**, 148–149 (2011).
4. Tegos, G., Stermitz, F. R., Lomovskaya, O. & Lewis, K. Multidrug pump inhibitors uncover remarkable activity of plant antimicrobials. *Antimicrob. Agents Chemother.* **46**, 3133–3141 (2002).
5. Aiello, D. *et al.* Discovery, characterization and comparison of inhibitors of Bacillus anthracis and Staphylococcus aureus replicative DNA helicases. *Bioorg. Med. Chem.* **17**, 4466–4476 (2009).
6. Opperman, T. J. *et al.* Aryl rhodanines specifically inhibit staphylococcal and enterococcal biofilm formation. *Antimicrob. Agents Chemother.* **53**, 4357–4367 (2009).
7. Gonzalez, B. E. *et al.* Severe Staphylococcal sepsis in adolescents in the era of community-acquired methicillin-resistant Staphylococcus aureus. *Pediatrics* **115**, 642–648 (2005).
