# Supplementary material for: trans-Translation inhibitors bind to a novel site on the ribosome and clear Neisseria gonorrhoeae in vivo
Source: Nat Commun. 2021 Mar 19;12:1799. doi: 10.1038/s41467-021-22012-7 (PMC7979765; doi:10.1038/s41467-021-22012-7)
Supplement: Supplementary file 2 — Description of Additional Supplementary Files [file 41467_2021_22012_MOESM2_ESM.pdf]

Description of Additional Supplementary Files

File Name: Supplementary Movie 1
Description: Change in position of the N terminus of ribosomal protein L27.
