# Supplementary material for: trans-Translation inhibitors bind to a novel site on the ribosome and clear Neisseria gonorrhoeae in vivo
Source: Nat Commun. 2021 Mar 19;12:1799. doi: 10.1038/s41467-021-22012-7 (PMC7979765; doi:10.1038/s41467-021-22012-7)
Supplement: Supplementary file 4 — Reporting Summary [file 41467_2021_22012_MOESM4_ESM.pdf]

nature research

Corresponding author(s): Kenneth Keiler & Christine Dunham

Last updated by author(s): Feb 12, 2021

Reporting Summary

Nature Research wishes to improve the reproducibility of the work that we publish. This form provides structure for consistency and transparency in reporting. For further information on Nature Research policies, see our Editorial Policies and the Editorial Policy Checklist.

Statistics

For all statistical analyses, confirm that the following items are present in the figure legend, table legend, main text, or Methods section.

n/a	Confirmed	
[ ]	[x]	The exact sample size (*n*) for each experimental group/condition, given as a discrete number and unit of measurement
[ ]	[x]	A statement on whether measurements were taken from distinct samples or whether the same sample was measured repeatedly
[ ]	[x]	The statistical test(s) used AND whether they are one- or two-sided<br>*Only common tests should be described solely by name; describe more complex techniques in the Methods section.*
[x]	[ ]	A description of all covariates tested
[x]	[ ]	A description of any assumptions or corrections, such as tests of normality and adjustment for multiple comparisons
[ ]	[x]	A full description of the statistical parameters including central tendency (e.g. means) or other basic estimates (e.g. regression coefficient) AND variation (e.g. standard deviation) or associated estimates of uncertainty (e.g. confidence intervals)
[ ]	[x]	For null hypothesis testing, the test statistic (e.g. *F*, *t*, *r*) with confidence intervals, effect sizes, degrees of freedom and *P* value noted<br>*Give P values as exact values whenever suitable.*
[x]	[ ]	For Bayesian analysis, information on the choice of priors and Markov chain Monte Carlo settings
[x]	[ ]	For hierarchical and complex designs, identification of the appropriate level for tests and full reporting of outcomes
[x]	[ ]	Estimates of effect sizes (e.g. Cohen's *d*, Pearson's *r*), indicating how they were calculated

Our web collection on statistics for biologists contains articles on many of the points above.

Software and code

Policy information about availability of computer code

Data collection	Leginon 3.2 National Resource for Automated Molecular Microscopy Automated transmission electron microscope image collection

Data analysis

Prism 8 and 9 GraphPad Data plotting and analyses, nonlinear regression
Excel 16 Microsoft Data analyses, tables
Appian 3.2 National Resource for Automated Molecular Microscopy Cryo-electron microscopy micrograph data pre-processing MotionCorr
Motioncorr2 Regents of the University of California Micrograph motion correction
CTFFIND4 4 University of Massachusetts Medical School Contrast transfer function parameter estimation
GCTF 1.06 MRC Laboratory of Molecular Biology Contrast transfer function parameter estimation
DoG Picker 3.2 SBGrid Consortium Autopick particles
Find EM N/A MRC Laboratory of Molecular Biology Template based autopicking particles
RELION-3 3.0 MRC Laboratory of Molecular Biology Cryo-electron microscopy 3D volume refinements and classifications
PHENIX 1.17.1 PHENIX Industrial Consortium Map sharpening, model refinement and validation
3DFSC Processing server 3 New York Structural Biology Center; Salk Institute for Biological Studies Plotting directional 3D FSC and global resolution graphs
Bsoft 2.0.4 National Institutes of Health Local resolution estimation
Chimera 1.15 Regents of the University of California Structure analysis, figure making
COOT 0.9.2 MRC Laboratory of Molecular Biology Model building
ChimeraX 0.93 Regents of the University of California Structure analysis, figure making
PyMOL 2.1 Schrbdinger Structure analysis, figure making
Phoenix WinNonlin 8.3 Certara computation of pharmacokinetics parameters

For manuscripts utilizing custom algorithms or software that are central to the research but not yet described in published literature, software must be made available to editors and reviewers. We strongly encourage code deposition in a community repository (e.g. GitHub). See the Nature Research guidelines for submitting code & software for further information.

Data

Policy information about availability of data

All manuscripts must include a data availability statement. This statement should provide the following information, where applicable:

- Accession codes, unique identifiers, or web links for publicly available datasets
- A list of figures that have associated raw data
- A description of any restrictions on data availability

Source data for Figs. 1, 2, 4, Supplementary Fig. 1, and Supplementary Tables 1-7 are provided at this link. Structural data have been deposited as PDB accession code: 6OM6, https://www.rcsb.org/structure/unreleased/60M6; EM accession code: EMD-20121 https://www.ebi.ac.uk/pdbe/entry/emdb/EMD-20121. All other data are included in this published article and its supplementary information files, or are available from the authors on request.

Source data for Figs. 1, 2, 4, Supplementary Fig. 1, and Supplementary Tables 1-7 are provided at this link. Structural data have been deposited as PDB accession code: 6OM6, https://www.rcsb.org/structure/unreleased/60M6; EM accession code: EMD-20121 https://www.ebi.ac.uk/pdbe/entry/emdb/EMD-20121. All other data are included in this published article and its supplementary information files, or are available from the authors on request.

Field-specific reporting

Please select the one below that is the best fit for your research. If you are not sure, read the appropriate sections before making your selection.

☒ Life sciences ☐ Behavioural & social sciences ☐ Ecological, evolutionary & environmental sciences

For a reference copy of the document with all sections, see nature.com/documents/nr-reporting-summary-flat.pdf

Life sciences study design

All studies must disclose on these points even when the disclosure is negative.

Sample size	Based on historical data from our laboratory, we need 5 mice per group to detect a significant difference (p = 0.044) of our positive control antibiotic (gentamicin) compared to PBS provided the 80% of the PBS control group stays colonized for 8 days. The difference is nonsignificant if we use 4 mice per group. However, 5 mice per group has been insufficient to detect efficacy for test compounds that did not clear as effectively as GEN, even with 100% of the PBS control group remaining infected (p = 0.051). For these compounds, 8-10 mice per group are needed to detect a difference at the level of p < 0.05 when 80-84% of mice are colonized.
Data exclusions	No data were excluded from analysis.
Replication	The experiment was done twice and were two independent experiments.
Randomization	Mice were chosen randomly for drug or vehicle treatment. These were the only groups used.
Blinding	One person performed both the treatments and colony counting, and mice were tracked using cage numbers and tail colors.

Reporting for specific materials, systems and methods

We require information from authors about some types of materials, experimental systems and methods used in many studies. Here, indicate whether each material, system or method listed is relevant to your study. If you are not sure if a list item applies to your research, read the appropriate section before selecting a response.

Materials & experimental systems

n/a	Involved in the study
☒	☐ Antibodies
☐	☒ Eukaryotic cell lines
☒	☐ Palaeontology and archaeology
☐	☒ Animals and other organisms
☒	☐ Human research participants
☒	☐ Clinical data
☒	☐ Dual use research of concern

Methods

n/a	Involved in the study
☒	☐ ChIP-seq
☒	☐ Flow cytometry
☒	☐ MRI-based neuroimaging

Eukaryotic cell lines

Policy information about cell lines

Cell line source(s)	Caco-2 (ATCC), HepG2 (ATCC), HeLa (ATCC}
Authentication	none of the cell lines were authenticated before the experiment
Mycoplasma contamination	HeLa cells were checked for Mycoplasma contamination using MycoAlert Mycoplasma Detection Kit; other cell lines were not checked
Commonly misidentified lines (See ICLAC register)	no commonly misidentified lines were used

Animals and other organisms

Policy information about studies involving animals; ARRIVE guidelines recommended for reporting animal research

Laboratory animals	Female CD-1 mice 4-6 weeks old (Neosome}, female BALB/cAnNCr mice 6-7 weeks old (USU}
Wild animals	none
Field-collected samples	none
Ethics oversight	All animal experiments were conducted at the Uniformed Services University of the Health Sciences, a facility fully accredited by the Association for the Assessment and Accreditation of Laboratory Animal Care, under a protocol that was approved by the university's Institutional Animal Care and Use Committee.<br>For Neosome experiments, all procedures described are in compliance with the Animal Welfare Act, the Guide for the Care and Use of Laboratory Animals, and the Office of Laboratory Animal Welfare. These studies conform to the NeoSome IACUC policies and Operational Guidelines as approved by the IACUC membership, NeoSome Safety Officers, or Attending Veterinarian.

Note that full information on the approval of the study protocol must also be provided in the manuscript.
